# Kirigami‐Cut Pattern Designs for the Enhancement of Sensing Performance Induced by Programmable Out‐of‐Plane Deformation

**DOI:** 10.1002/advs.76637

**Published:** 2026-07-14

**Authors:** Xiaodong Huang, Yuhao Wu, Shan Lu, Liguo Qin, Xin Ge, Guangneng Dong, Qiao Hu

**Affiliations:** ^1^ Key Laboratory of Education Ministry For Modern Design and Rotor‐Bearing System Institute of Design Science and Basic Components School of Mechanical Engineering Xi'an Jiaotong University Xi'an P. R. China; ^2^ Department of Materials‐Oriented Chemical Engineering School of Chemical Engineering Fuzhou University Fuzhou P. R. China; ^3^ School of Mechanical Engineering and Shaanxi Key Laboratory of Intelligent Robots Xi'an Jiaotong University Xi'an P. R. China

**Keywords:** kirigami pattern, meta‐structure, pressure sensor, PVDF

## Abstract

Kirigami meta‐structures are popular for their excellent mechanical properties including high stretchability, multi‐stable states and programmable deformation. Recent studies have integrated sensing material with kirigami meta‐structure to develop strain sensor with high stretchability or good surface conformability. This study reports on three types of kirigami patterns, and utilizes the programmable out‐of‐plane displacements of kirigami meta‐structure in couple with polyvinylidene fluoride (PVDF) film to construct piezoelectric pressure sensor with enhanced sensing performance, such as sensitivity and output voltage. The basic kirigami patterns and the parameter design methods are discussed. Then, the kirigami pattern is fabricated on PVDF films using nanosecond‐pulse ultraviolet laser in couple with craft. Experimental results in combination with simulation analysis confirm the deformation characteristics of kirigami‐cut PVDF. Finally, the deformed kirigami‐cut PVDF is embedded into polydimethylsiloxane (PDMS) to construct piezoelectric pressure sensor, output signal measurements confirm enhanced sensing output compared to sensors implanted with un‐deformed kirigami‐cut PVDF, with an increase of approximately 1.76 times. Theoretical analysis reveals the improvement of force transmission efficiency induced by deformed kirigami‐cut structure. The study findings validate the potential of kirigami meta‐structures as a scalable and versatile foundation for next‐generation pressure sensor design.

## Graphical abstract

Different kirigami patterns are proposed and the parameters are optimized by comparing the out‐of‐plane deformation behavior. Then, the proposed patterns are fabricated on PVDF film using laser technology. The deformed kirigami‐cut PVDF is embedded into PDMS to construct pressure senor. Experimental results prove that the introduction of deformed kirigami‐cut structure improves force transmission efficiency, and thus enhancing sensor performance.

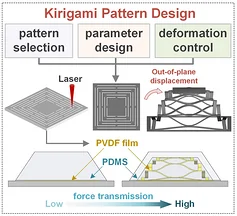

## 1 Introduction

Recently, mechanical meta‐structures based on kirigami have aroused extensive research interest [1, 2, 3]. Due to the special structural design, kirigami meta‐structure exhibits excellent mechanical properties such as super‐stretchablity [4, 5, 6], programmable deformation [7, 8, 9, 10, 11], multi‐stability [12], etc. Typical kirigami meta‐structures consist of a series of periodic pattern cuts, which could induce out‐of‐plane buckling or in‐plane rotation, thus leading to multifunctional three‐dimensional structures, realizing exceptional mechanical properties. Hence, these meta‐structures have potential applications in many fields including stretchable electronic devices [13, 14], soft robotics [15, 16], and biomedical engineering [2, 17, 18].

Piezoelectric materials can convert external mechanical stimulus into electrical responses [19]. Piezoelectric sensors have been extensively used for detecting forces, deformations and vibrations. Although various approaches have been devised to enhance the sensor performance, such as fabricating microstructures on the substrates [20], improving the electrical property of the material [21, 22], or changing the sensory layer from 2D to 3D [23], the sensor performance remains limited. To further improve the piezoelectrical performance, engineering solutions utilizing kirigami technology have attracted a lot of attention.

Over all, the integration of kirigami structures with piezoelectric material involves two ways [24], one is to utilize kirigami meta‐structures as structural substrates to enhance the surface conformability of e‐skins [25, 26, 27], especially the utilization of closed‐loop kirigami, which features an outside‐in distribution of self‐similar pattern cuts [24, 28]. For instance, Hong et al. reported an ultra‐stretchable kirigami piezo‐metamaterials which could sense in‐ and out‐of‐plane coupled large deformations with up to 200% strains [14]. Besides, Li et al. presented a tower‐inspired 2D kirigami patterns, which could be stretched elastically into a 3D kirigami structure [29]. The other one involves fabricating kirigami structures on sensor sheets to directly improve the sensor performance in a more effective manner under various deformations [14, 30, 31, 32]. For example, Song et al. developed a stretchable strain sensors using a kirigami‐cut polyvinylidene fluoride (PVDF) film, achieving a stretchability of up to 400% through the structural distortion of the cut parts [30]. Similarly, Kim et al. combined experiments and simulations to analyze the electromechanical properties of sensors with different kirigami patterns and get the optimized kirigami patterns [33].

However, all these researches concentrated on the high stretchability induced by kirigami cuts to enhance the sensor performance of strain senor. The multi‐stability and programmability of kirigami meta‐structure are often neglected [14], especially for closed‐loop kirigami, the out‐of‐plane deformation can be adjusted and thus leading to stable deformed structure with different displacements, the programmable deformed structure exhibits great potential in the sensor structure design.

Here, based on multi‐stability and programmability of closed‐loop kirigami, we report on a series of kirigami pattern designs. First, through numerical simulation, the deformation behavior and stress analysis were compared between the proposed kirigami patterns. Besides, we proposed several key parameters to expand the application of designed patterns, and concluded the design procedure of kirigami patterns according to the requirements. Furthermore, by cutting the pattern on the PVDF film, kirigami‐cut PVDF films coupled with different out‐of‐plane deformations were created. Notably, through a combination of experiments and finite element simulations, great consistencies are obtained in terms of the deformation behavior of kirigami‐cut PVDF. Finally, the practical application of kirigami‐cut patterns was demonstrated through embedding the deformed kirigami‐cut PVDF film into polydimethylsiloxane (PDMS) with the shape of semi‐pyramid to construct a piezoelectric pressure sensor, simulation results and sensor output analysis confirmed that the pressure sensor with deformed kirigami‐cut PVDF exhibited enhanced voltage output compared to sensor with un‐deformed kirigami‐cut PVDF. These results testify that the deformed kirigami‐cut structure could enhance force transmission efficiency, and thus improving the sensor output performance. This study highlights the potential of kirigami‐cut structures to enhance the sensor performance by improving force transmission efficiency due to the various deformations.

## 2 Results and Discussion

### 2.1 Concept and Design

Figure 1 presents the design of the basic kirigami patterns. As shown in the figure, all three basic patterns exhibit a shape‐shifting behavior, transforming from 2D sheet to 3D structure. Differently, Pattern X (Figure 1a) was designed to be centrally symmetric, which would lead to out‐of‐plane deformation (*u_y_* = *H*), while Pattern Z (Figure 1b) was set to be rotationally symmetric, resulting in rotation of central square (*r_y_* = *θ*) in addition to out‐of‐plane deformation (*u_y_* = *H*), hence, leading to two different pattern deformation results, namely Pattern Z and Pattern ZwR (Figure 1c). All these patterns can transform from planar state to three‐dimensional state, maintaining stable at each displacement. This characteristic offers many possibilities for further applications.

**FIGURE 1:**
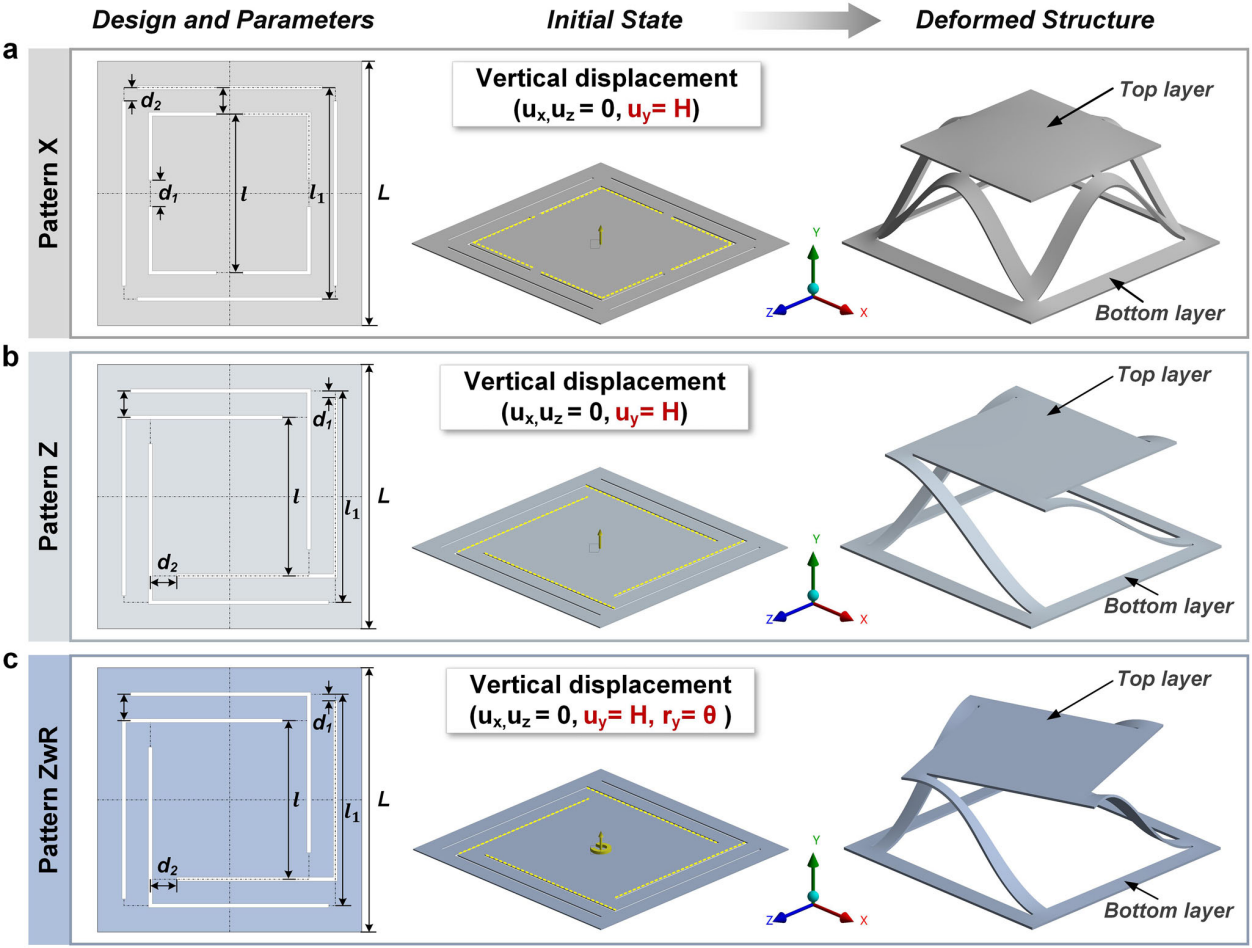
Concept design and the deformation effect of the kirigami patterns. Schematic illustrations of kirigami pattern design and deformation results of (a) Pattern X, (b) Pattern Z and (c) Pattern ZwR.

For each unit pattern, it contains a total of 5 designed parameters (*L*, *D_1_*, *D_2_*, *d_1_*, *d_2_*, where *l_1_* = *L – D_1_, l* = *l_1_ – D_2_*), as depicted in Figure 1. *L* and *l* are determined by actual application requirements, while *d_1_* and *d_2_* would influence the upper limit of out‐of‐plane deformation. The pattern structure can be further optimized through changing these design variables according to the application requirement. Meanwhile, the upper limit of different patterns is different, which depends on the deformation characteristics of the pattern. Hence, the analysis of deformation characteristics of different kirigami patterns is of great importance.

### 2.2 Deformation Analysis and Structural Characteristics

To compare the deformation behaviors of different patterns, finite element analysis (FEA) was conducted using commercial software (Ansys 2022 R1). The parameters for each pattern case are listed in Table S1 to specify the analysis conditions. In order to compare the deformation of different patterns, some structural parameters were fixed, including *L*, *D_1_* and *D_2_*. Since the pattern was set to be applied on PVDF films, the material properties used in FEA are based on the PVDF bought from Jinzhou Kexin Electronic Material Co., Ltd. Mechanical properties of PVDF are listed in Table S2. The boundary conditions employed in the simulation are shown in Figure S1, four edges in the bottom layer were fixed, while four edges in the top layer were applied with out‐of‐plane displacement (*H*). Especially, the rotation in Y axis of Pattern ZwR model was set free, which would cause the rotation of central square in addition to the out‐of‐plane displacement.

As shown in Figure S2, with the same out‐of‐plane deformation of *H* = 8 mm, the stress distributions of different pattern models were different. High stress area concentrated at the shear hinge, there are eight stress concentration areas for each pattern model. Due to the structure design, the concentration area of Pattern X involves two high stress points, while the concentration area of Pattern Z and Pattern ZwR involves only one high stress point. A quantitative comparison shows that the average stress (σ_avg_) of Pattern X (16.229 MPa) is 3.38 times higher than that of Pattern Z (4.4794 MPa), and 2.59 times higher than that of Pattern ZwR (6.2589 MPa), indicating that under same deformation, Pattern X exhibits better response compared to Pattern Z and Pattern ZwR, and the stress distribution of Pattern X is centrally symmetric while the stress distribution of Pattern Z and Pattern ZwR is rotationally symmetric. This is consistent with the pattern design.

To further compare the deformation behaviors of different patterns, the average stress (σ_avg_) and maximum stress (σ_max_) of each pattern models as a function of deformation (*H*) are calculated through simulation. As depicted in Figure 2, σ_avg_ and σ_max_ increase with *H*, and this trend was observed in all pattern models. However, the increase rate (Δσ_avg_/Δ*H*) of Pattern X was 2.029, much larger than that of Pattern Z (0.560) and Pattern ZwR (0.819), meaning that before reaching the yield strength of the material, Pattern X is a better choice because of the larger increase rate. While in some situation, Pattern Z is better because it can achieve a higher H without reaching the yield strength. In all, each pattern has its advantages, researchers can choose corresponding patterns according to their needs. For instance, when it is applied to the sensor design, Pattern X would be the top choice because it could achieve higher sensitivity compared to the other two.

**FIGURE 2:**
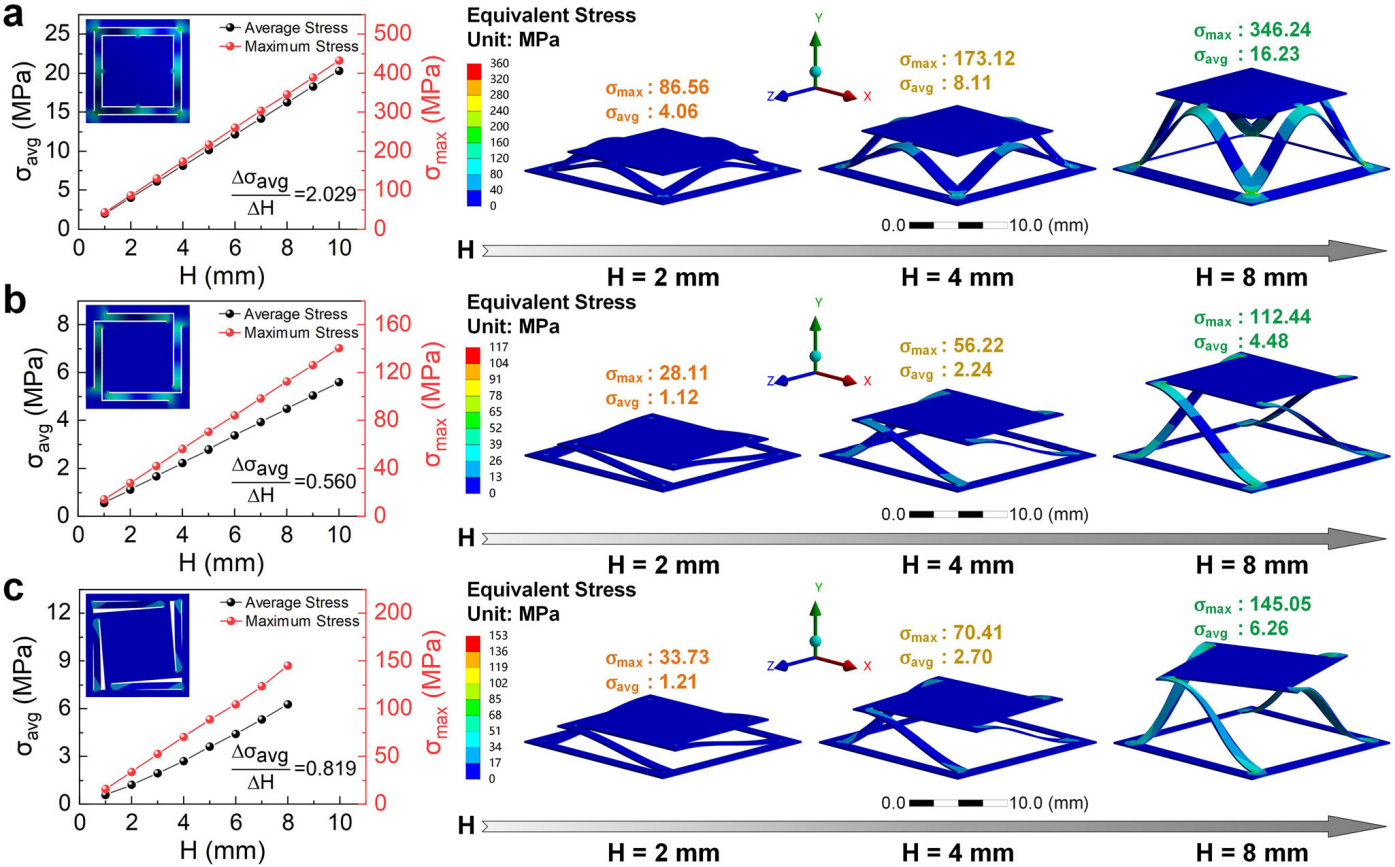
Deformation characteristics and Stress analysis of the kirigami patterns. Average and maximum stress as a function of deformation (H) of (a) Pattern X, (b) Pattern Z and (c) Pattern ZwR.

### 2.3 Multi‐Application Enabled by Designed Patterns

The single unit of different patterns has been discussed above, however, in practical implementation, the application of designed patterns is very complex and diverse. As illustrated in Figure 3, three key variables were put forward for the utilization of designed kirigami patterns: the number of sides (*N_s_*), the number of pattern units (*N_p_*) and the relative direction of out‐of‐plane deformation (i.e., same or reverse).

**FIGURE 3:**
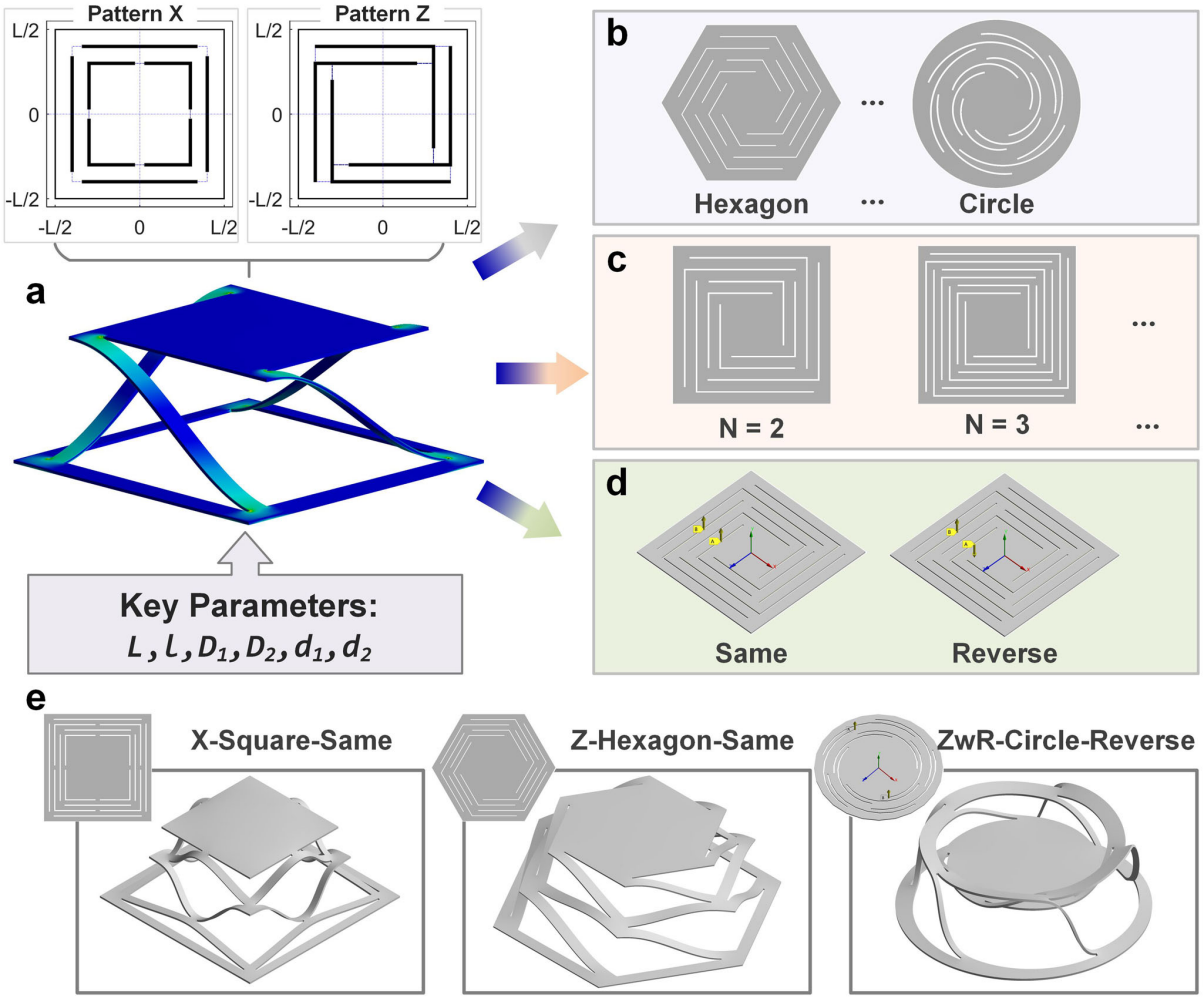
The application of kirigami patterns and the key variables. (a) Schematic illustrations of single pattern and the deformation. Key variables to expand the application of kirigami patterns such as (b) The number of sides (*N_s_*), (c) The number of layers (*N_p_*) and (d) The direction of out‐of‐plane deformation compared to the previous layer. (e) Typical application cases by changing these key variables.

First, by increasing *N_s_*, the pattern can be applied to different shape, from quadrangle, hexagon to circle, indicating that the shape of PVDF films used can be chosen according to the application scenarios (Figure 3b).

Second, *N_p_* is in direct relation to the maximum displacement of the kirigami pattern. However, the augmentation in *N_p_* of patterns results in a substantial reduction in the top layer area, leading to a pyramid outline (Figure 3c).

Third, the direction of out‐of‐plane deformation can be changed. Take for example a two‐layer pattern, the direction of out‐of‐plane deformation of the second layer can be the same as that of first layer, or conversely, as shown in Figure 3d. The introduction of out‐of‐plane deformation direction would further expand its application scenarios, such as the combination of PVDF sheet with other type of sensors. Figures S3–S5 specifically illustrate the deformation results by changing the key variables of Pattern X, Pattern Z and Pattern ZwR, respectively.

The application of designed kirigami patterns usually involves multiple pattern units (*N_p_>2*), in that case, structure parameters (*D_i_*) become extreme important, since they would influence the final deformed state. To study the effect of *D_i_* on the deformation states of our designed kirigami patterns, the simulation of a three‐layer four‐sided PVDF film with different types of *D_i_* was conducted.

After confirming the following parameters such as *L = 20 mm*, *l = 8 mm*, *N_p =_ 3*, and *N_s_* = 4, three types of *D_i_* were proposed. As shown in Figure 4a, *D_i_* of Type 01 was set to be a constant, *D_i_* in Type 02 constructs arithmetic progression, while *D_i_* in Type 03 is geometric progression, specific values and their relationship can be seen in Figures S6 and S7, and Table 1. The deformation values of each layer and the average stress were calculated through FE analysis. Figure 4b,c displays the deformation of each layer, *H_3rd_* of all cases are the same, since the analysis setting of displacement of top layer was set to be 8 mm. However, *H_1st_* and *H_2nd_* are different because of pattern structure and parameter type. Specifically, for each parameter type, regular response was discovered: *H_1st_* and *H_2nd_* of Pattern X are the smallest, while *H_1st_* of Pattern ZwR is the biggest, *H_2nd_* of Pattern Z is the biggest. The value of *H_1st_*, *H_2nd_* and *H_3rd_* would finally determine the shape of envelope surface of deformed 3D structure. For each pattern, the deformation response of Type 01 is the biggest, and this is constant with all pattern types. Take Pattern X for example, *H_1st_* of Type 01 is the biggest, and *H_2nd_* of Type 01 is also the biggest. Interestingly, *H_1st_* of Type 02 (3.3323 mm) is bigger than that of Type 03 (3.1552 mm), *H_2nd_* of Type 02 (6.6692 mm) is also bigger than that of Type 03 (6.5078 mm).

**FIGURE 4:**
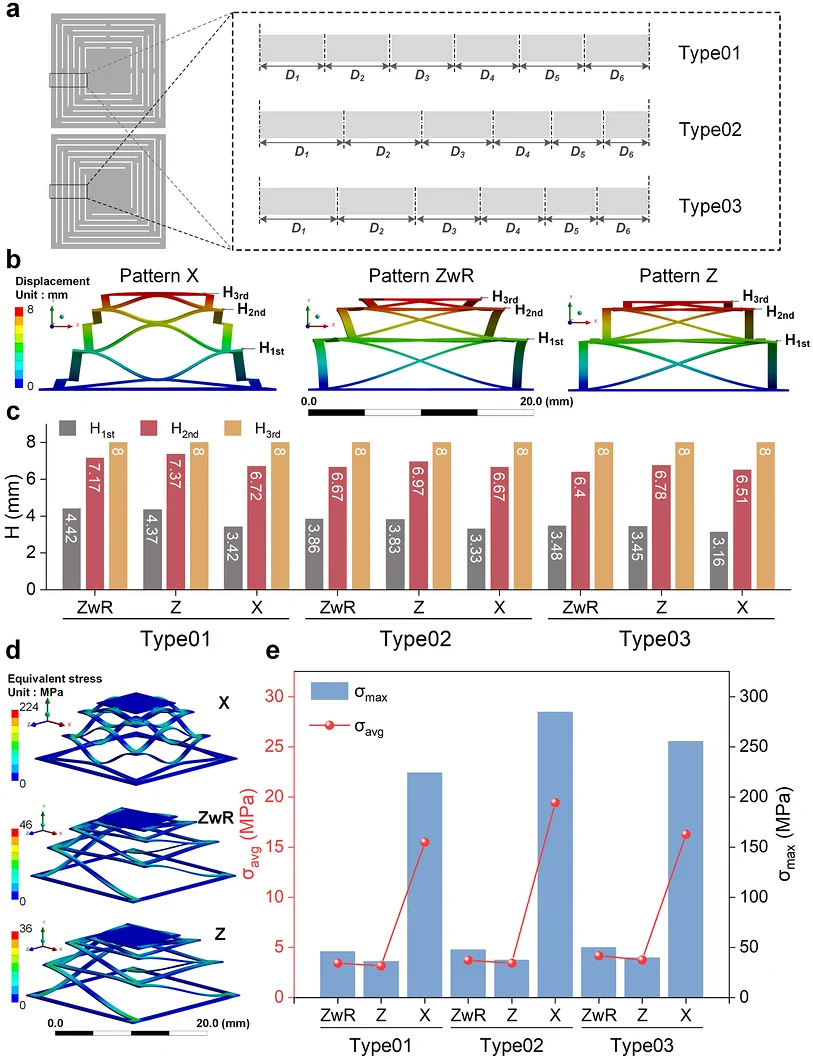
Deformation and stress response of different pattern cuts along with different types of parameter. (a) Schematics of three types of kirigami structures parameters, namely Type01, Type02 and Type03, along with their corresponding parameters. (b) Deformation response of different type of kirigami pattern designs, namely Pattern X, Pattern ZwR and Pattern Z. (c) Numerical simulation results of the deformation of different pattern cuts along with different parameters. (d) Stress distribution of different type of kirigami pattern designs, namely Pattern X, Pattern ZwR and Pattern Z. (e) Numerical simulation results of the average stress of different pattern cuts along with different parameters.

**TABLE 1:** Design parameters of each metamaterial model for parametric studies.

Pattern	Cases	Design parameters [mm]										
		*L*	*l*	*N*	*d_1_*	*d_2_*	*D_1_*	*D_2_*	*D_3_*	*D_4_*	*D_5_*	*D_6_*
**Pattern X**	**1**	20	8	3	0.8	0.8	1.0	1.0	1.0	1.0	1.0	1.0
	**2**	20	8	3	0.8	0.8	1.125	1.075	1.025	0.975	0.925	0.875
	**3**	20	8	3	0.8	0.8	1.316	1.316	0.9696	0.9696	0.7144	0.7144
**Pattern Z**	**1**	20	8	3	0	1.0	1.0	1.0	1.0	1.0	1.0	1.0
	**2**	20	8	3	0	1.025	1.125	1.075	1.025	0.975	0.925	0.875
	3	20	8	3	0	0.9696	1.316	1.316	0.9696	0.9696	0.7144	0.7144

Figure 4d,e presents the stress distribution and average stress results of different patterns. Although parameter types change the value of average stress of patterns, the basic trend of average stress change of different patterns hasn't been changed, *σ_avg_* of Pattern X is always the biggest, and *σ_avg_* of Pattern Z is always the smallest. However, the effects of parameter types on pattern types are different, specifically, take Pattern X for example, *σ_avg_* of type 01 is 15.515 MPa, *σ_avg_* of type 02 is 19.43 MPa, *σ_avg_* of type 03 is 16.262 MPa, meaning that the improving effects of parameter types on Pattern X is the biggest, up to 25.23%.

Generally speaking, pattern type is the top key factor for application. After that, parameter type will also influence the deformation behavior of the kirigami pattern, but the effects on different pattern types are different. These findings mean that before determining specific parameters, pattern types should be the top consideration. Hence, we summarized the process of pattern application as follows (Figure S8):

Step 1. Specify the application scenario, and choose the corresponding pattern type;
Step 2. Decide the basic parameters, such as *L*, *d_1_*, *d_2_, l*, *N_s_*;
Step 3. Choose the optimal *N_p_* by comparing the *σ_avg_* of the deformed pattern according to the simulation results;
Step 4. Get the optimal *D_i_* types by evaluating the outer contour of deformed patterns;
Step 5. Summarize all the parameters and establish the kirigami pattern.

### 2.4 Practical Demonstration of Kirigami‐Cut PVDF Films

The designed kirigami patterns were proposed to be applied on PVDF films, so as to get deformed kirigami‐cut PVDF films to be further applied in the sensor design. The pattern fabrication process was illustrated in Figure 5a, a polarized PVDF film coated with Al electrode was purchased (Jinzhou Kexin Electronic Material Co., Ltd) and used in the fabrication process. A nanosecond‐pulse ultraviolet laser was used to fabricate the kirigami pattern and remove the Al electrode layer, then the PVDF layer was cut into designed patterns using a craft cutter. Since the designed cut width was 200 µm, we compared several laser fabrication parameters such as mark loop, speed, laser power and frequency, after comparing the measured width under optical microscope (Figure S9), the optimal fabrication parameters was finally decided as shown in Figure 5b.

**FIGURE 5:**
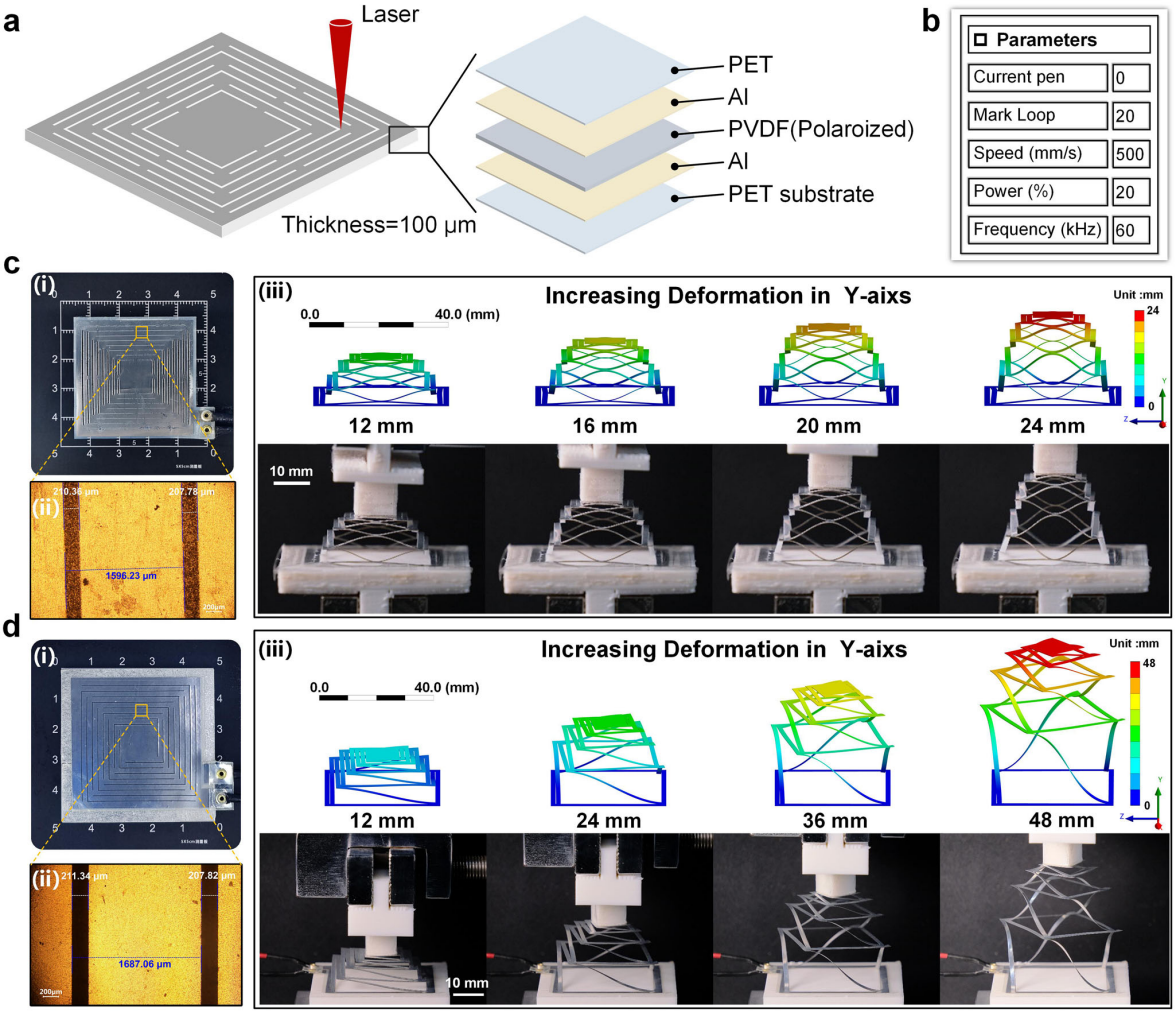
Fabrication of designed kirigami‐cut patterns on PVDF film and the deformation results. (a) Laser cutting process and layer structure of the PVDF film. (b) Fabrication parameters used in the laser cutting process. (c) Photograph and optical microscope picture of fabricated PVDF film and comparison of deformation behavior of kirigami‐cut PVDF film with Pattern X in the FE models and experiments. (d) Photograph and optical microscope picture of fabricated PVDF film and comparison of deformation behavior of kirigami‐cut PVDF film with Pattern ZwR in the FE models and experiments.

To demonstrate the deformation analysis of the kirigami‐cut PVDF films, two different patterns were designed and fabricated on PVDF films, then the deformation behaviors of fabricated kirigami‐cut PVDF films were compared between experiments and numerical simulations. The deformation responses of kirigami‐cut PVDF films are studied under various pullout displacement applied at the center by conducting tensile test on a cyclic tensile testing machine. Numerical models are developed using ANSYS 2022R1 to investigate the mechanical behaviors (coupled large deformation). The PVDF films used have a size of 40 cm × 40 cm and a thickness of 100 µm.

Figure 5c displays the out‐of‐plane deformation of kirigami‐cut PVDF film with a four‐sided four‐layered Pattern X. As shown in Figure 5c(i),(ii), the measured width is 210.36 µm, and the machining error is around 5.18%, indicating that the fabricated kirigami pattern conforms to the initial design. As the out‐of‐plane deformation reaches 12, 16, 20, 24 mm, the deformation of kirigami‐cut PVDF film in the simulation is highly consistent with that of experiment(Figure 5c(iii)).

The consistency between experiments with numerical simulation results was also testified on kirigami‐cut PVDF film with a four‐sided four‐layered Pattern ZwR. According to Figure 5d(i),(ii), the proposed pattern was successfully fabricated on the PVDF film. Since the deformation limit of Pattern ZwR is higher than that of Pattern X, the deformation states of kirigami‐cut PVDF film with a four‐sided four‐layered Pattern ZwR under out‐of‐plane displacements from 12 to 48 cm were analyzed. The high consistency between experiments with numerical simulation further validate the reliability of the simulation data, indicating that the simulation results are reliable and can be used to analyze the deformation behavior of kirigami‐cut PVDF with the same type of kirigami patterns.

The deformation behavior comparisons between experiments and numerical simulations indicates that the proposed kirigami patterns can be perfectly fabricated on PVDF films, and the deformation behavior of kirigami‐cut PVDF film is highly consistent with our design and expectation.

After ensuring the deformation characteristics of kirigami‐cut PVDF film, we proposed to embed the kirigami‐cut PVDF film into PDMS to construct a piezoelectric pressure sensor. We believe that compared to un‐deformed PVDF film, sensor implanted with deformed PVDF film exhibits higher performance since it could change the force transmission structure, thus improving force transmission efficiency.

To testify this hypothesis, numerical simulation and experiment are conducted to compare the properties of sensors implanted with deformed PVDF film with different pattern cuts. As shown in Figure 6a, the displacement of kirigami‐cut PVDF film was set to be 0 mm, 6 mm with pattern Z, and 6 mm with pattern X, namely SensorH0, SensorH6‐Z, and SensorH6‐X. The size of kirigami‐cut PVDF film was 20 × 20 mm^2^, and the thickness is 100 µm. Specific parameters of designed kirigami patterns used are shown in Figures S10 and S11, and the fabricated kirigami‐cut PVDF films show high consistency with initial design. After ensuring the design of kirigami pattern, the deformed kirigami‐cut PVDF films are embedded into PDMS with the shape of semi‐pyramid to construct different pressure sensors. The designed parameters of constructed sensor are shown in Figure S12.

**FIGURE 6:**
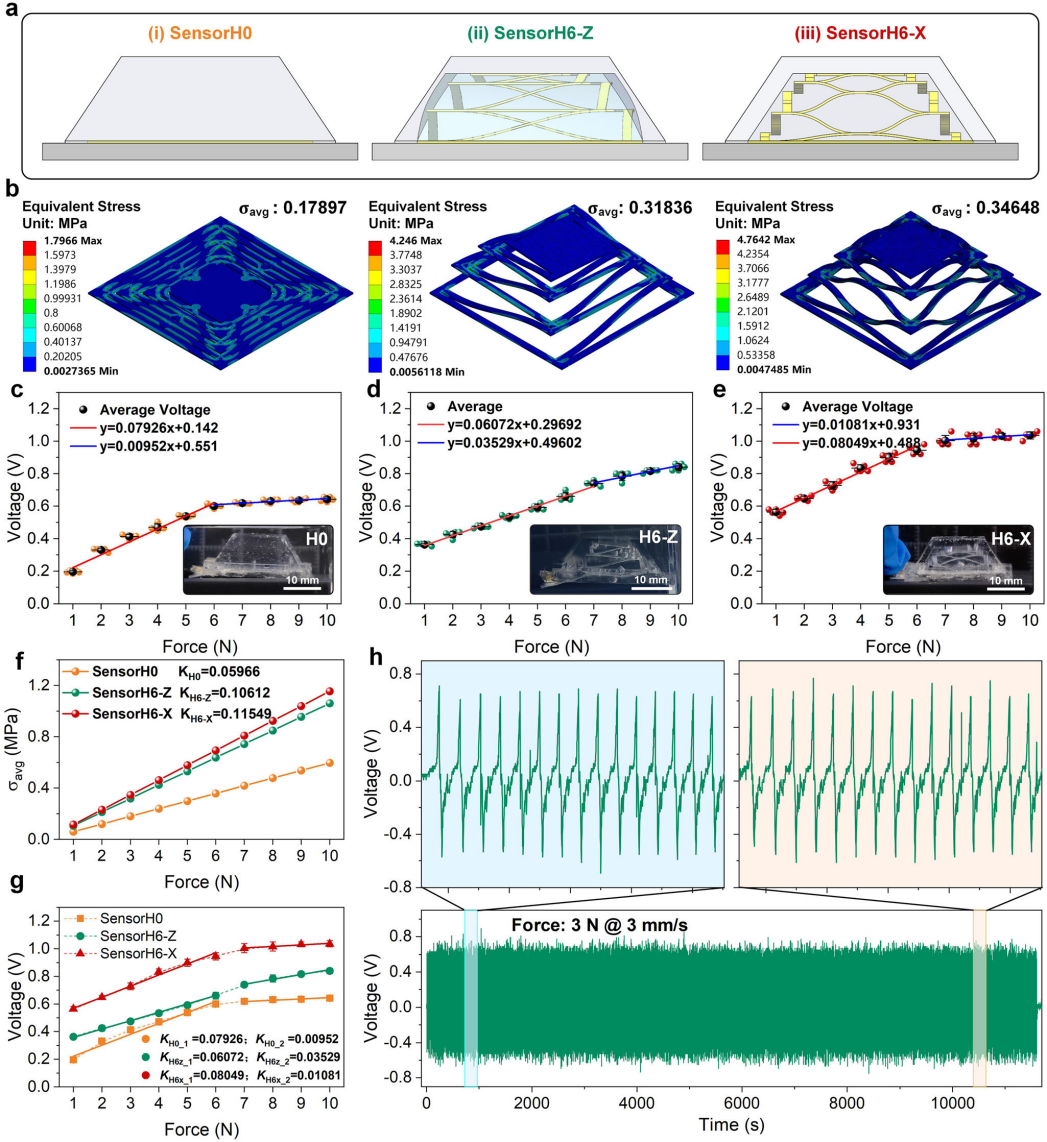
The comparisons of sensing performance between sensors implemented with deformed kirigami‐cut PVDF films with different patterns. (a) Schematic illustrations of sensors implemented with deformed kirigami‐cut PVDF films with displacement of 0 mm (SensorH0), 6 mm with pattern Z (SensorH6‐Z), and 6 mm with pattern X (SensorH6‐X). (b) Stress distributions of SensorH0, SensorH6‐Z and SensorH6‐X according to FEA. Measured output voltage as a function of applied normal force of (c) SensorH0, (d) SensorH6‐Z and (e) SensorH6‐X. The loading speed is 3 mm/s. (f) Numerical simulation results of SensorH0, SensorH6‐Z and SensorH6‐X. (g) Comparison of output voltage of different sensors as a function of applied normal force. (h) Long‐term durability test of SensorH6‐X over 10 000 loading cycles under force of 3 N at the loading speed of 3 mm/s.

Numerical simulation (see *Materials and Methods 4.3* in detail) results are shown in Figure 6b, under the same loading force of 3 N, the stress distribution and average stress of PVDF film are calculated to compare the stress transferred to the film. Compared to that of SensorH0 (0.17897 MPa), the average stress (*σ_avg_*) of deformed kirigami‐cut PVDF is much higher, as the *σ_avg_* of SensorH6‐Z is 0.31836 MPa, and *σ_avg_* of SensorH6‐X is 0.34648 MPa. This indicates that the deformed kirigami‐cut pattern designs effectively improved the stress transferred to the PVDF film. Besides, the enhancement effect of pattern X is larger than that of pattern Z, and this is consistent with previous simulation results. To further testify our hypothesis, *σ_avg_* of different sensors as a function of loading force from 1 to 10 N was also calculated. As shown in Figure 6f, at the same load, *σ_avg_* of SensorH6‐Z and SensorH6‐X is always bigger than that of SensorH0, and *σ_avg_* increases with the value of displacements of deformed kirigami‐cut PVDF. Further analysis indicates that the rate of change (σ_avg_/Δ*F*) of SensorH6‐X is 0.11549, bigger than that of SensorH6‐Z (0.10612), and both of these two are bigger than that of SensorH0 (0.05966). The simulation results indicate that sensor implanted with deformed kirigami‐cut PVDF shows a higher force transmission efficiency, leading to a higher σ_avg_ and larger rate of change (σ_avg_/Δ*F*).

Apart from the numerical simulation, sensors embedded with deformed kirigami‐cut PVDF films were fabricated to further testify our hypothesis. Specific fabrication process can be seen in *Materials and Methods 4.4* in detail. And the final fabricated sensors are shown in Figure 6c–e, side view of the sensor clearly demonstrated the deformation effect of kirigami‐cut PVDF film in PDMS, the deformation status of kirigami‐cut PVDF film is highly consistent with previous simulation results. After that, sensor output voltage was measured through an oscilloscope. Specific signal acquisition process can be seen in *Materials and Methods 4.5* in detail. The voltage output as a function of load was measured by cyclic loading process, the average of five data points was calculated. Averaged voltage output as a function of loading force from 1 to 10 N was depicted in Figure 6g, there are same trends with these three sensors. For each sensor, the voltage‐force curve can be divided into two stages, the sensitivity, which can be defined as Δ*V*/Δ*F*, of the first stage is much bigger than that of second stage, we deduced that this is mainly due to the stiffening effect of soft PDMS, the deformation of PDMS bump was continuously attenuated with increasing pressure, leading to a decrease in sensitivity in the second stage. Besides, under same normal force, the output voltage of SensorH6‐X is bigger than that of SensorH6‐Z, and that of these two are both bigger than that of SensorH0, meaning that the introduction of deformed kirigami‐cut structure did increase the output voltage of pressure sensor. Furthermore, for the first stage, the sensitivity of SensorH0 is 0.07926 V/N, while the sensitivity of SensorH6‐Z is 0.06072 V/N, both of these are smaller than that of SensorH6 (0.08049 V/N). The comparison of sensitivity indicates that the deformed kirigami‐cut PVDF didn't increase the sensitivity significantly. Comprehensively considering the output voltage and sensitivity obtained through linear fitting calculation, we could find that although the implantation of deformed kirigami‐cut PVDF didn't change sensor sensitivity significantly, it did increase the sensor output voltage greatly in the low‐force area, indicating that the implantation of deformed kirigami‐cut PVDF improved the force transmission efficiency, resulting in a bigger output voltage in the low‐force stage. Besides, the signal amplitude and waveform fidelity are consistently maintained throughout 10000 loading cycles under force of 3 N (Figure 6h and Figure S15), confirming its mechanical durability and signal stability during long‐term operation.

Furthermore, to study the effect of out‐of‐plane deformation on the performance of sensor, sensor implanted with deformed PVDF film with pattern X and displacement of 4 mm, namely SensorH4‐X (Figure S16a), was fabricated and tested. As shown in Figure S16b,c, with the same pattern X, as the deformation increases, the value of σ_avg_ rises correspondingly according to the simulation results. The piezoelectric performance of SensorH4‐X was measured and analyzed (Figure S16d). The comparison of sensing performance between SensorH4‐X and SensorH6‐X indicates that the enhancement effect of force transmission efficiency increases with out‐of‐plane displacements of kirigami‐cut PVDF with pattern X.

Both experimental findings and numerical simulation results testify that the introduction of deformed kirigami‐cut structure on PVDF film greatly improves the force transmission efficiency, and thus leading to a higher sensor output. This is consistent with our initial hypothesis. As summarized in Table S4, compared to other structure designs for the enhancement of piezoelectric sensing performance, our design to introduce deformed kirigami‐cut structure for the improvement of force transmission efficiency is novel and effective, and we believe that this method will provide guidance on the sensor structure design in the future.

## 3 Conclusions

In this study, a series of kirigami patterns that can maintain multi‐stable deformation states were developed. Previous study focused on the high stretchability of kirigami structure to develop strain sensors with ultrahigh stretchability, which is essential in many wearable sensor applications. However, this study concentrated on the multi‐stable states of the kirigami pattern, by altering the out‐of‐plane deformation of designed kirigami patterns to construct different types of pressure sensor.

First, three types of basic patterns were proposed, namely Pattern X, Pattern Z and Pattern ZwR. The deformation behavior and stress analysis were compared between different patterns through numerical simulation. Each pattern has its unique advantages, meaning that the kirigami pattern can be chosen according to the application scenarios.

Second, to extend the application of kirigami pattern, several parameters were put forward, *N_p_*, *N_s_* and the direction of out‐of‐plane deformation. For each pattern, the above parameters could be changed to expand the application of kirigami patterns. Besides, we put forward three types of *D_i_* and compared their deformation characteristics and stress conditions. Furthermore, the general procedure of designing kirigami pattern based on the application requirements was summarized.

Thirdly, a four‐side three‐layer kirigami pattern was applied on PVDF films to construct a piezoelectric pressure sensor. The fabrication of kirigami patterns on PVDF film can be made cost‐effective with laser processing and craft cutting. After that, the deformation of kirigami‐cut PVDF was detected through numerical simulation and experiments. In addition, the effects of kirigami‐cut PVDF on the sensor performance were analyzed. The sensing performance of the sensors embedded with kirigami‐cut PVDF with different patterns were compared in terms of output voltage, sensitivity, and linearity. Experimental results indicate that deformed kirigami‐cut structure improves force transmission efficiency, and thus increasing the output voltage of the sensor. Moreover, theoretical analysis was performed, which proved the dependence of improvement effects of deformed kirigami‐cut structure on force transmission efficiency.

This work systematically proposed three types of basic kirigami patterns and the key structural parameters in terms of its deformation behavior and application expansion. After that, based on the proposed application procedure, a four‐side three‐layer Pattern X kirigami pattern was designed to construct pressure tactile sensor. Through numerical simulation and experiments, the hypothesis that deformed kirigami‐cut PVDF can improve force transmission efficiency, resulting in a higher sensor output was proved.

We believe that this study can guide the development of pressure tactile sensors, as the deformed kirigami‐cut structure provides new directions for the structure design of tactile sensor, and these sensors can be used in various applications, such as texture recognition. In addition, this sensor can be further developed by integration with other type of sensors such as piezoresistive sensor, since the deformed kirigami‐cut structure provides space for the integration of other type of sensitive layer.

## 4 Materials and Methods

### 4.1 Numerical Simulations for Kirigami‐Cut PVDF Deformation

The FEA simulations were performed using commercial simulation software (Ansys 2022R1) to analyze the deformation behavior and stress distribution of proposed kirigami patterns. The kirigami‐cut PVDF film (Young's modulus (*E*) = 2.5 GPa, Poisson's ratio (*ν*) = 0.35) was modeled. The thickness is 100 µm. The four edges of bottom layer are fixed, and the eight edges of top layer are applied with out‐of‐plane displacement (*H*).

### 4.2 Laser Fabrication Conditions Selection

Considering the designed cut width was 200 µm, employing appropriate laser fabrication parameters was crucial. Hence, different laser parameters such as mark loop, frequency, speed, and laser power were used to fabricate a rectangle with the width of 200 µm and the length of 8 mm. Then, the width of fabricated rectangle was measured through optical microscope. Each pattern was fabricated three times, and the average width and its standard deviation were calculated. As shown Figure S9, by comparing the average width, the final selected processing parameters are shown in Figure S9c.

### 4.3 Numerical Simulations for Sensor Comparison

The stress distribution and average stress was analyzed by FEA simulation using commercial simulation software (Ansys2022R1). Sensors embedded with kirigami‐cut PVDF film with different patterns and displacements were modeled, specific dimension parameters are shown in Figure S12. The mechanical properties of the materials were set as depicted in Table S3. The analysis settings are shown in Figure S14, the bottom surface of the sensor is set to be fixed, and top surface of structural steel is applied with normal pressure. The equivalent stress of deformed kirigami‐cut PVDF is obtained and analyzed.

### 4.4 Sensor Fabrication Process

The 3D‐printed template was cleaned with absolute alcohol, then the mixture of PDMS (Sylgard 184, Dow Corning Co., Ltd) with a mass ratio of prepolymer to curing agent at 10:1 was cast into the template. After curing at an oven (DZF‐6020AB, LICHEN Co., Ltd) at 60°C for 12 h, a semi‐pyramid‐shape PDMS contact layer was prepared. After that, the kirigami‐cut PVDF film was stick to the contact layer with the top layer stick to the top surface of the contact layer. Then the mixture of PDMS same as the last one was cast into the contact layer. The mold with mixture of PDMS was placed in an oven and cured at 60°C for 12 h to obtain the pressure sensor (see in Figure S13).

### 4.5 Experimental Setup and Testing

The voltage output of the piezoelectric pressure sensor was recorded by a digital oscilloscope (TBS 2104X Tektronix (China) Co., Ltd). For cyclic loading, the sensor was placed under a cylindrical probe, operated by an universal tensile testing machine, the cyclic loading procedure can be changed by adjusting the loading speed and peak force as shown in Figure S17. In the cyclic loading process, the loading speed was set to be 3 mm/s, the peak force was adjusted from 1 to 10 N, and output voltage was measured and recorded. As for the recorded data, the peak voltage was measured and the mean value was calculated, error bars were calculated using mean ± SD (standard deviation).

## Author Contributions

**Xiaodong Huang**: Conceptualization, Software, Investigation, Data Curation, Methodology, Writing – Original Draft. **Yuhao Wu**: Investigation, Data Curation. **Shan Lu**: Writing – Review & Editing, Data Curation. **Liguo Qin**: Conceptualization, Methodology, Writing – Reviewing and Editing, Funding acquisition. **Xin Ge**: Formal analysis, Supervision, Writing – Reviewing and Editing. **Guangneng Dong**: Writing – Review & Editing, Validation. **Qiao Hu**: Writing – Review & Editing, Resources, Funding acquisition.

## Conflicts of Interest

The authors declare no conflicts of interest.

## Supporting information




**Supporting File**: advs76637‐sup‐0001‐SuppMat.docx.

## Data Availability

All data generated or analyzed during this study are included in the published article. The data supporting the findings of this study are available from the corresponding author upon reasonable request.
