# Supplementary material for: Kirigami‐Cut Pattern Designs for the Enhancement of Sensing Performance Induced by Programmable Out‐of‐Plane Deformation
Source: Adv Sci (Weinh). 2026 Jul 14:e76637. Online ahead of print. doi: 10.1002/advs.76637 (PMC13367109; doi:10.1002/advs.76637)
Supplement: Supplementary file 1 — Supporting File: advs76637‐sup‐0001‐SuppMat.docx. [file ADVS-9999-e76637-s001.docx]

**Supplementary Materials**

Kirigami-cut Pattern Designs for the Enhancement of Sensing Performance Induced by Programmable Out-of-plane Deformation

***Xiaodong Huang ^a^, Yuhao Wu ^a^, Shan Lu ^a^,***

***Liguo Qin ^a,^ *,*** ***Xin Ge ^c,^ *, Guangneng Dong ^a^, Qiao Hu ^b,^ ****

**^a^** Key Laboratory of Education Ministry for Modern Design and Rotor-Bearing System, Institute of Design Science and Basic Components, School of Mechanical Engineering, Xi’an Jiaotong University, Xi’an 710049, P. R. China.

**^b^** School of Mechanical Engineering and Shaanxi Key Laboratory of Intelligent Robots, Xi’an Jiaotong University, Xi’an 710049, PR China.

**^c^** Department of Materials-Oriented Chemical Engineering, School of Chemical Engineering, Fuzhou University, Fuzhou, PR China

*****Corresponding author: [liguoqin@xjtu.edu.cn](mailto:liguoqin@xjtu.edu.cn) (Liguo Qin), hqxjtu@xjtu.edu.cn (Qiao Hu)

**Table S1**. Design parameters of each pattern model for parametric studies.

| **Types** | ***D_1_***/mm | ***D_2_***/mm | ***d_1_***/mm | ***d_2_***/mm | ***L***/mm |
| --- | --- | --- | --- | --- | --- |
| Pattern X | 1.5 | 1.5 | 0.8 | 0.8 | 20 |
| Pattern Z | 1.5 | 1.5 | 0 | 1.5 | 20 |
| Pattern ZwR | 1.5 | 1.5 | 0 | 1.5 | 20 |

**Table S2**. Mechanical properties of PVDF films used for parametric studies .

|  | **Density[kg/m^3^]** | **Poisson’s ratio** | **Young’s modulus[MPa]** | **Thickness[μm]** |
| --- | --- | --- | --- | --- |
| PVDF | 1780 | 0.35 | 2500 | 100 |


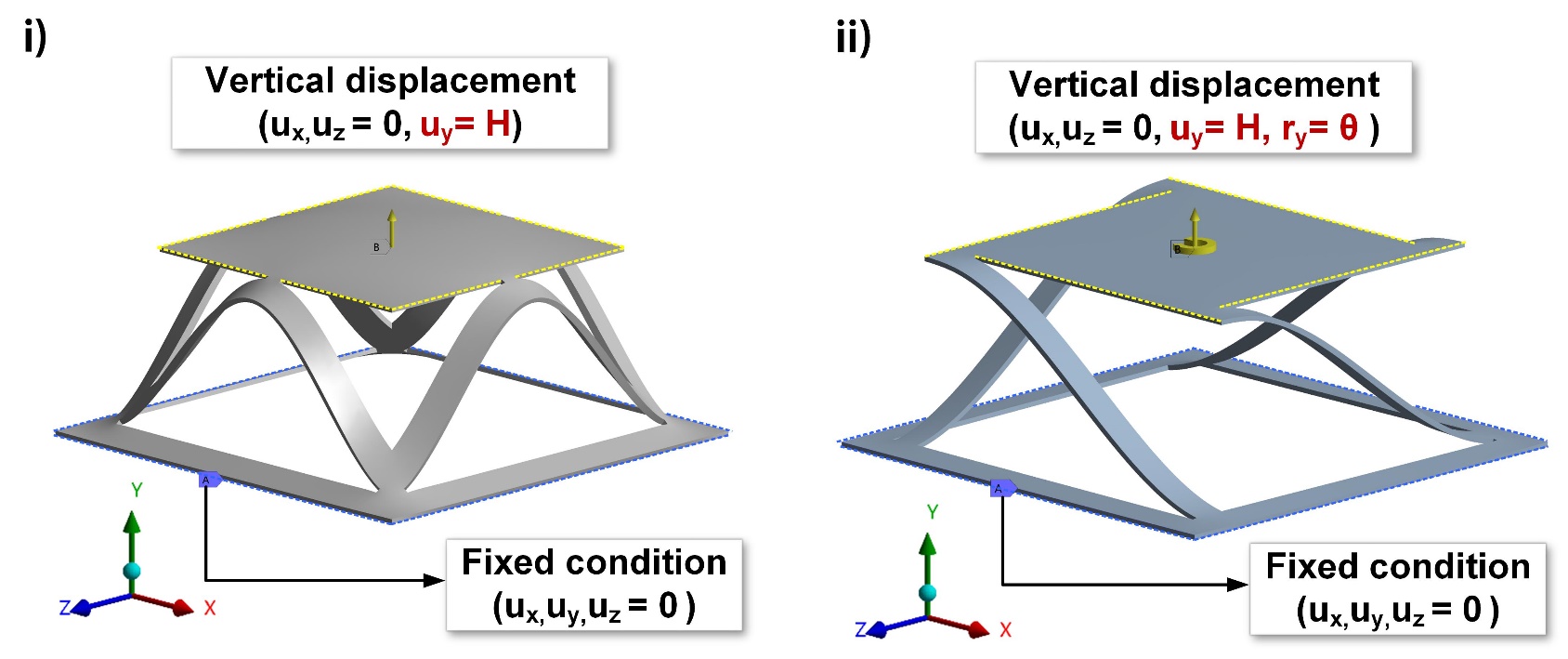


**Figure S1. Simulation settings of the kirigami patterns.** a) Pattern X , b) Pattern Z.


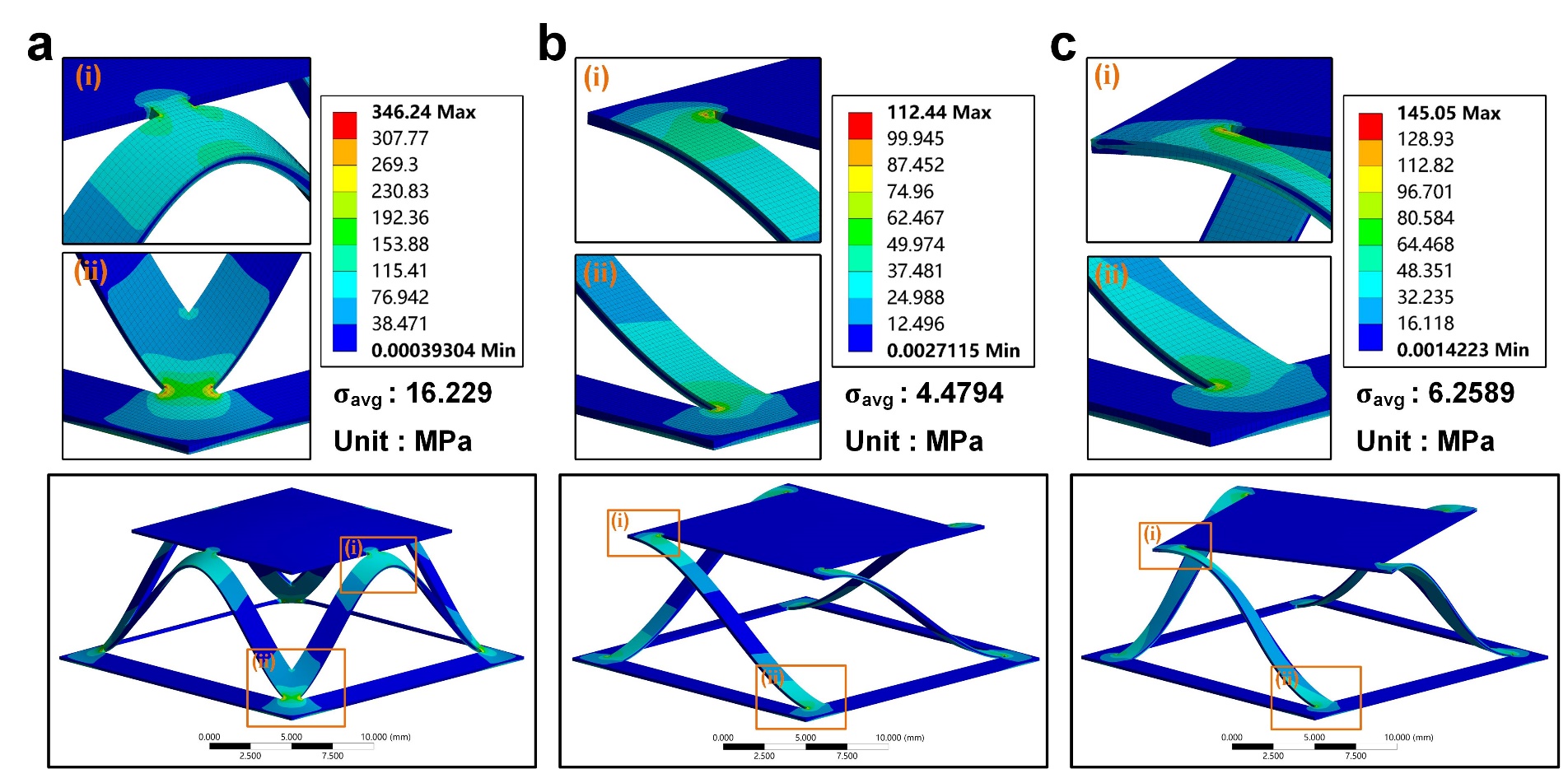


**Figure S2. Finite element analysis of the kirigami patterns.** Stress distribution and equivalent Stress of (a) Pattern X, (b) Pattern Z and (c) Pattern ZwR.


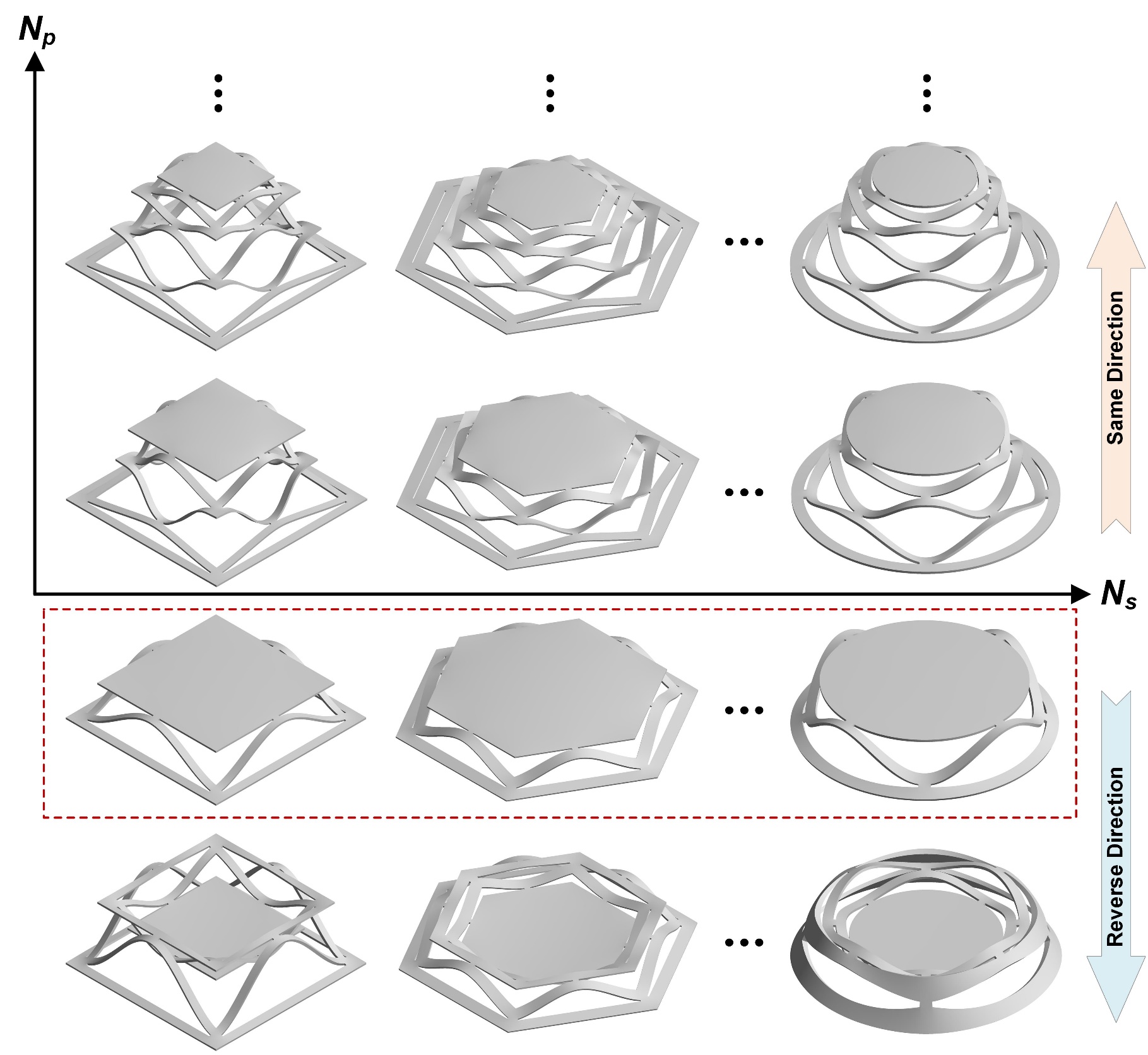


**Figure S3 . The deformation results by changing the key variables of Pattern X.**


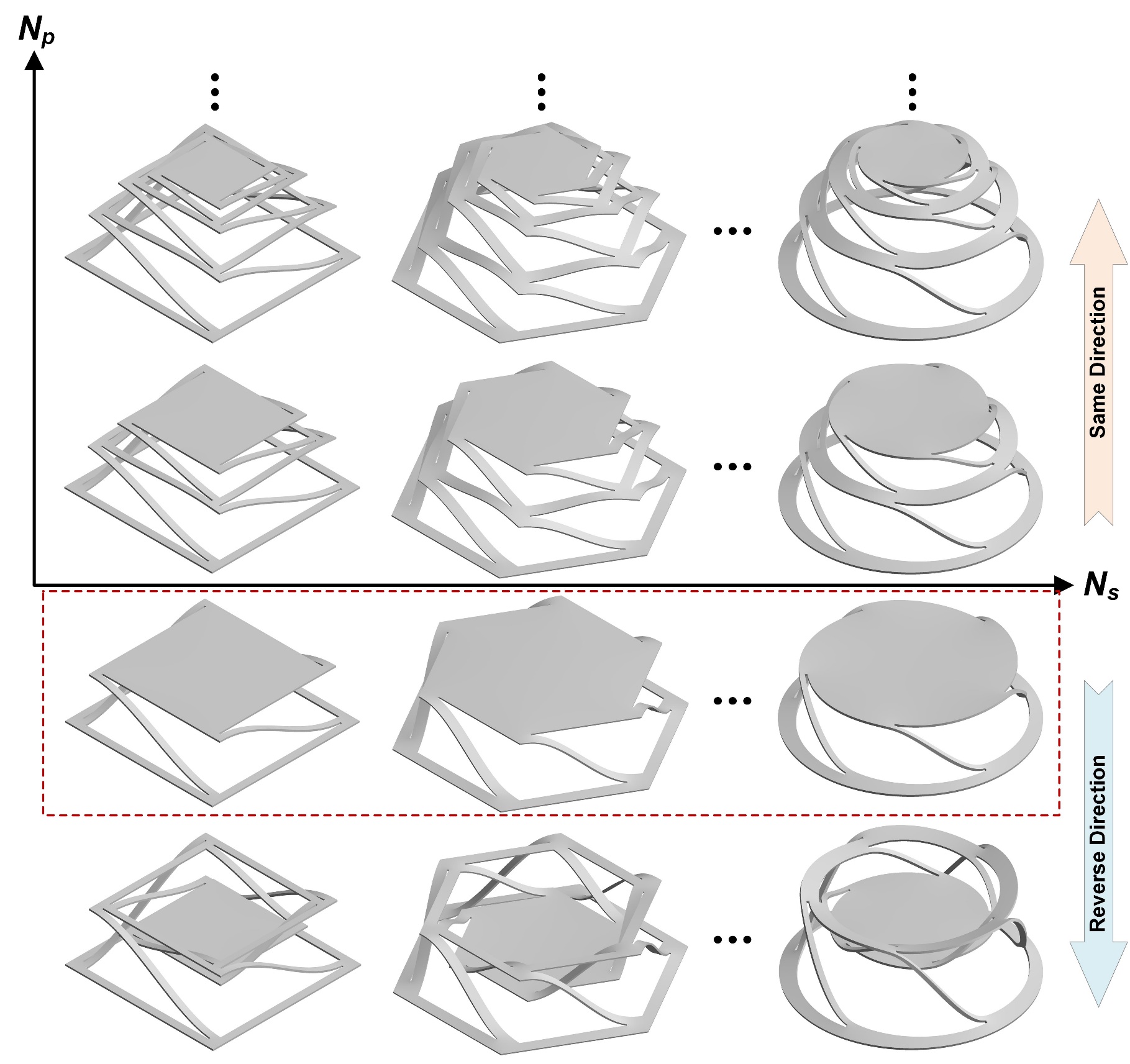


**Figure S4 . The deformation results by changing the key variables of Pattern Z.**


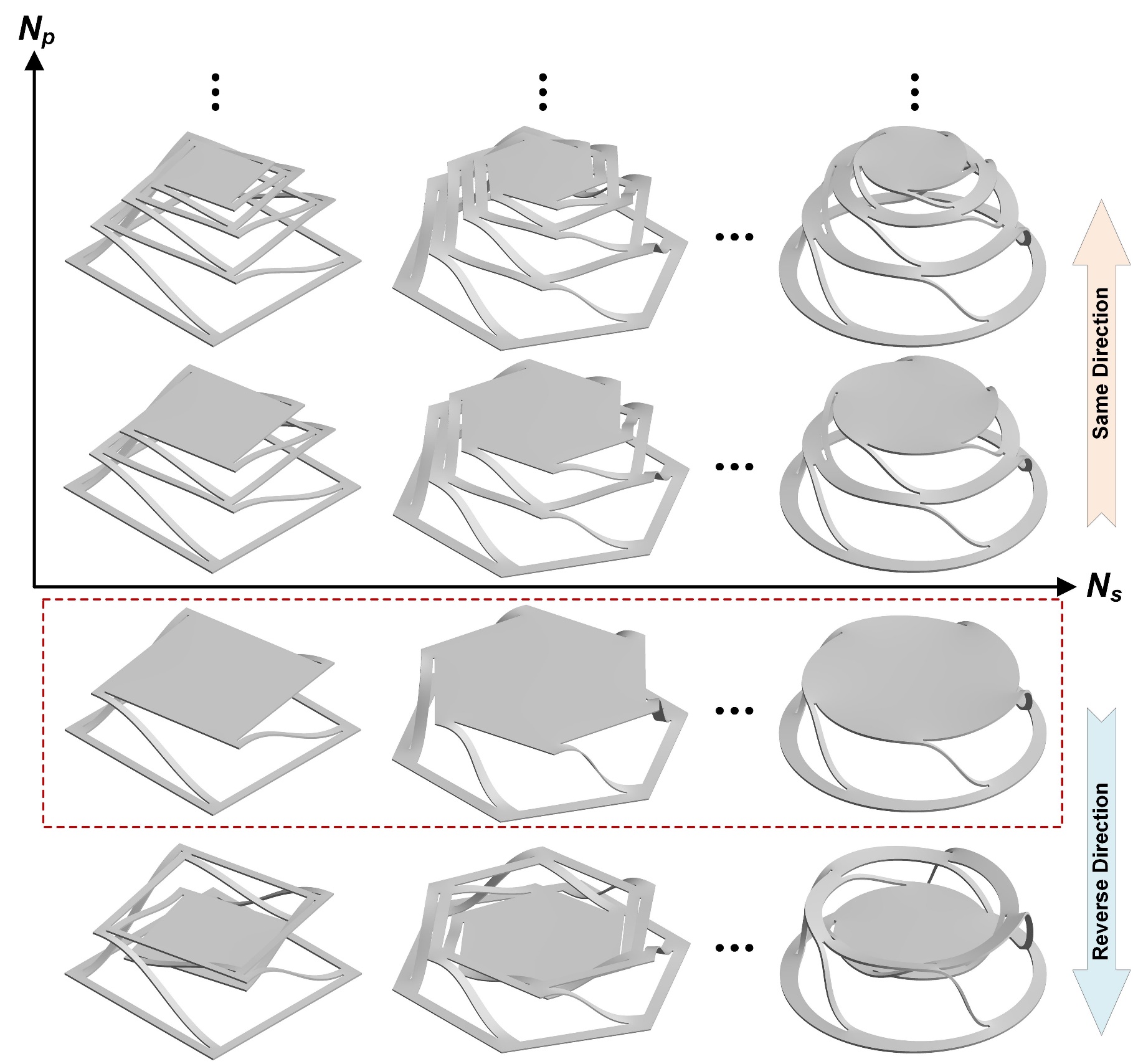


**Figure S5 . The deformation results by changing the key variables of Pattern ZwR.**


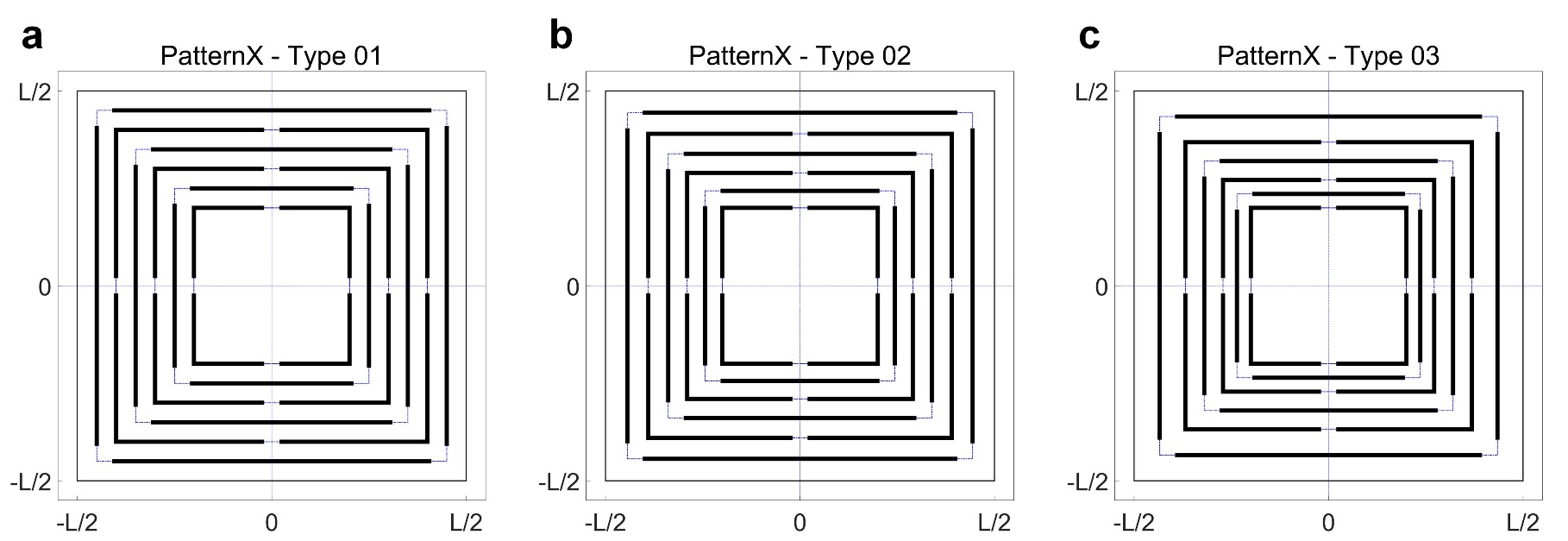


**Figure S6 . Schematics of three types structures of kirigami Pattern X.** a) Type 1 , b) Type 2, and c) Type 3.


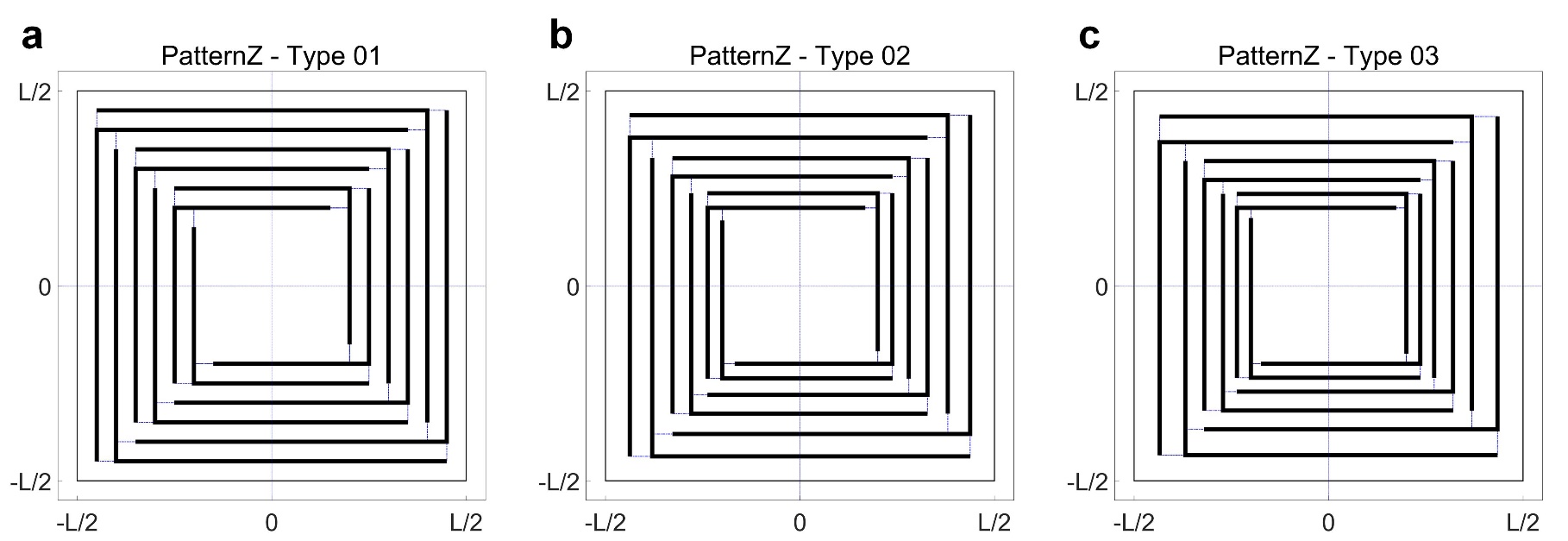


**Figure S7. Schematics of three types structures of kirigami Pattern Z.** a) Type 1 , b) Type 2, and c) Type 3.

In type 01, as depicted in **Figure S6(a)** and **Figure S7(a)**, *D_i_* was set to be a constant, calculated by the following equation:

$$D_{i}=const=\frac{L-l}{4N}$$

Type 02 was shown in **Figure S6(b)** and **Figure S7(b)**, *D_i_* was set to be an arithmetic progression, meaning that $D_{i}$ decreases with $i$, $D_{i}$ can be described as:

$$D_{i}=\frac{L-l-2N(2N-1)d}{4N}-\left( i-1 \right)d,d=0.1$$

Type 03 was shown in **Figure S6(c)** and **Figure S7(c)**, *D_i_* of each layer is equal, and *D_i_* of different is geometric progression, $D_{i}$ can be described as:

$$D_{2i-1}=D_{2i}=\frac{\left( 1-p \right)(L-l)}{4(1-p^{N})}p^{i-1},p=\log_{N} \frac{l}{L}$$

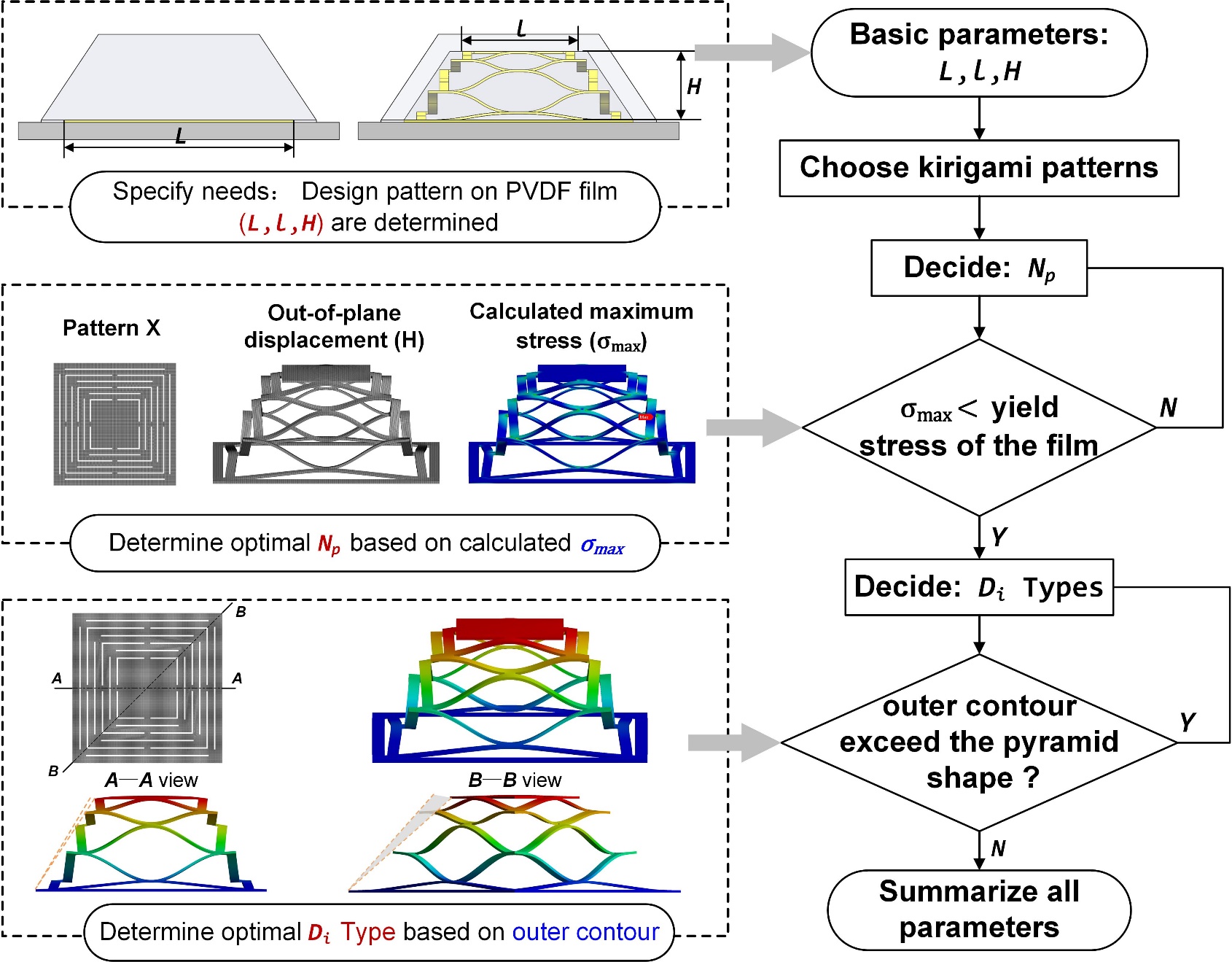


**Figure S8. Flowchart of the decision process of final kirigami pattern design according to the application scenario.**


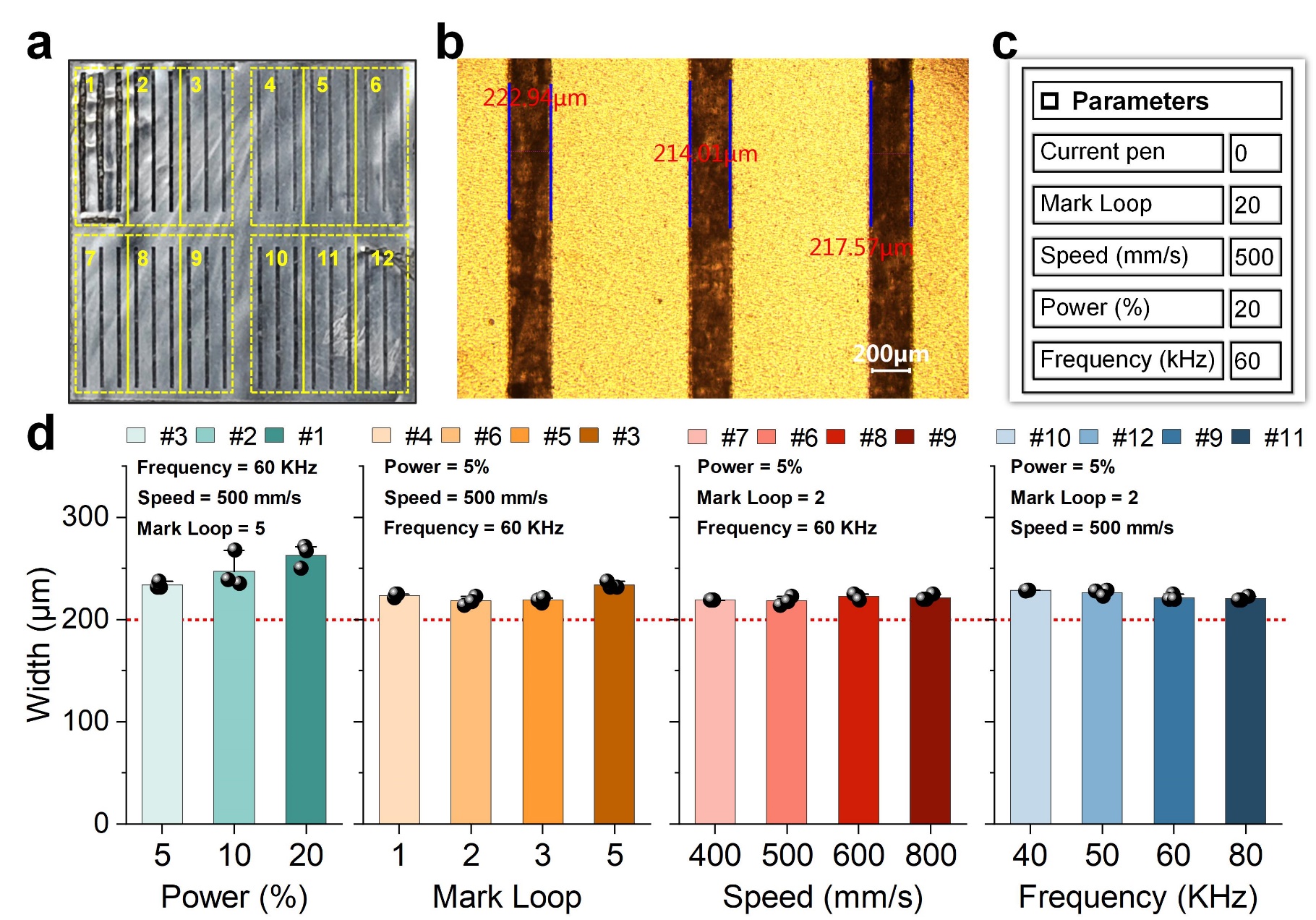


**Figure S9 . The comparison of laser fabrication parameters and measured width.**


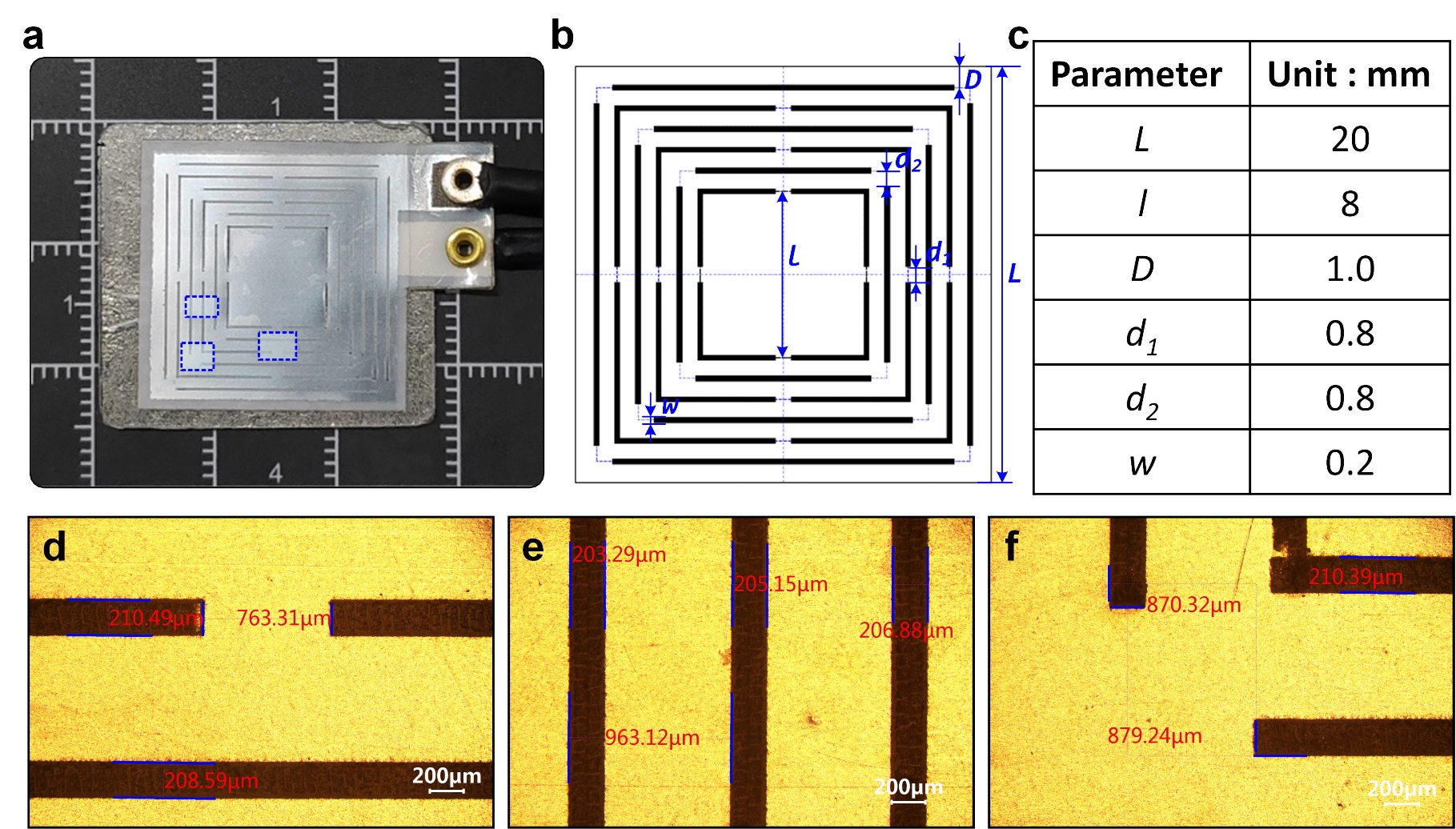


**Figure S10 . The photograph and optical microscope picture of kirigami-cut PVDF film with pattern X.**

**
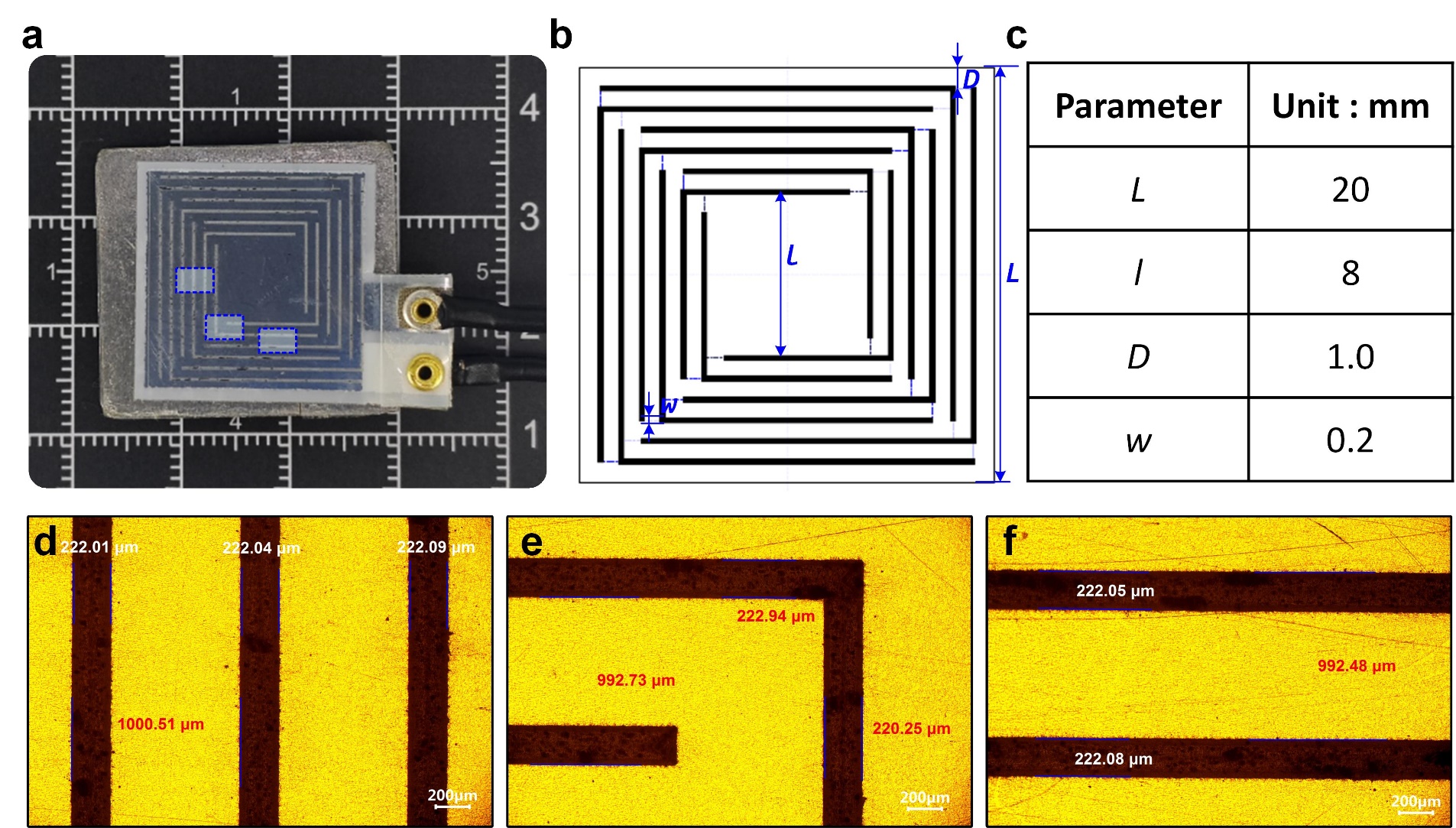
**

**Figure S11 . The photograph and optical microscope picture of kirigami-cut PVDF film with pattern Z.**


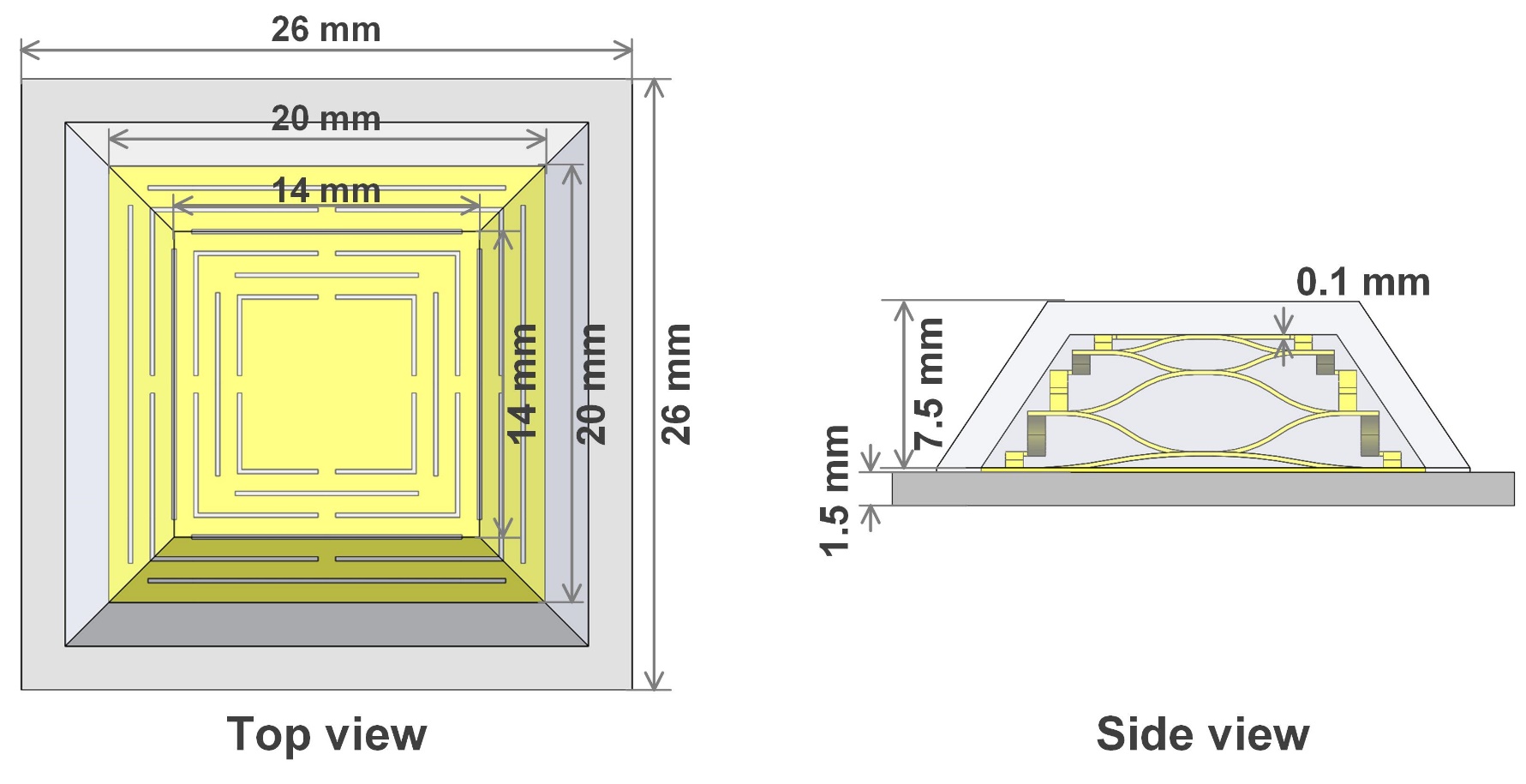


**Figure S12 . The size parameters of designed piezoelectric pressure sensor implanted with deformed kirigami-cut PVDF film.**


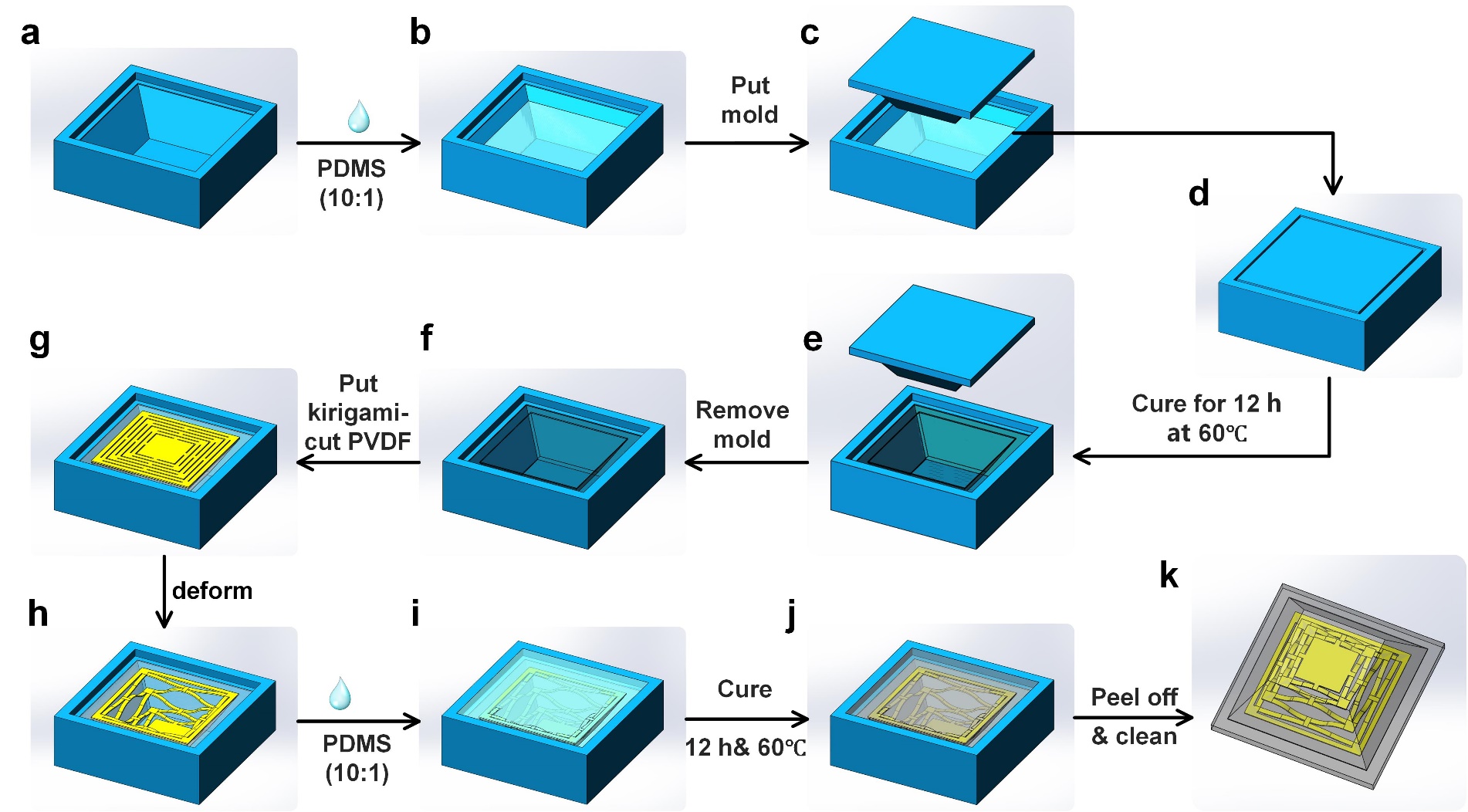


**Figure S13. Schematic drawing of the fabrication process of proposed piezoelectric pressure sensor.** (a). printed pit mold. (b) pit mold with PDMS solution. (c) pit mold and pillar mold on it. (d) curing procedure of contact layer. (e) remove of pillar mold. (f) assembling of the kirigami-cut PVDF film. (g) deform process of kirigami-cut PVDF film. (h) deformed kirigami-cut PVDF film in the mold. (i) new mold with PDMS solution. (j) curing procedure of bottom layer. (k) proposed piezoelectric pressure sensor.


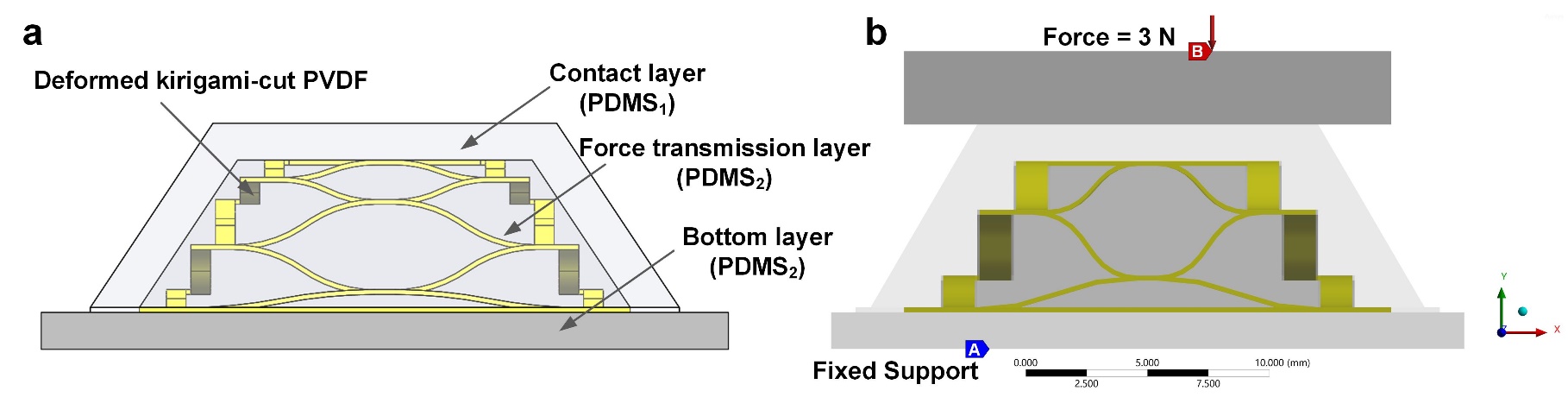


**Figure S14.** **(a)** The material types of each layer used in the simulation. **(b)** The schematics of sensor simulation settings.

**Table S3**. Mechanical properties of material used for FEA of sensor analysis.

| **Material** | **Density[kg/m^3^]** | **Poisson’s ratio** | **Young’s modulus** |
| --- | --- | --- | --- |
| PVDF | 1780 | 0.35 | 2500 MPa |
| Structural  Steel | 7850 | 0.3 | 200 GPa |
| PDMS_2_ | 971 | 0.46 | 1.5 MPa |
| PDMS_1_ | 971 | 0.46 | 2.2 MPa |


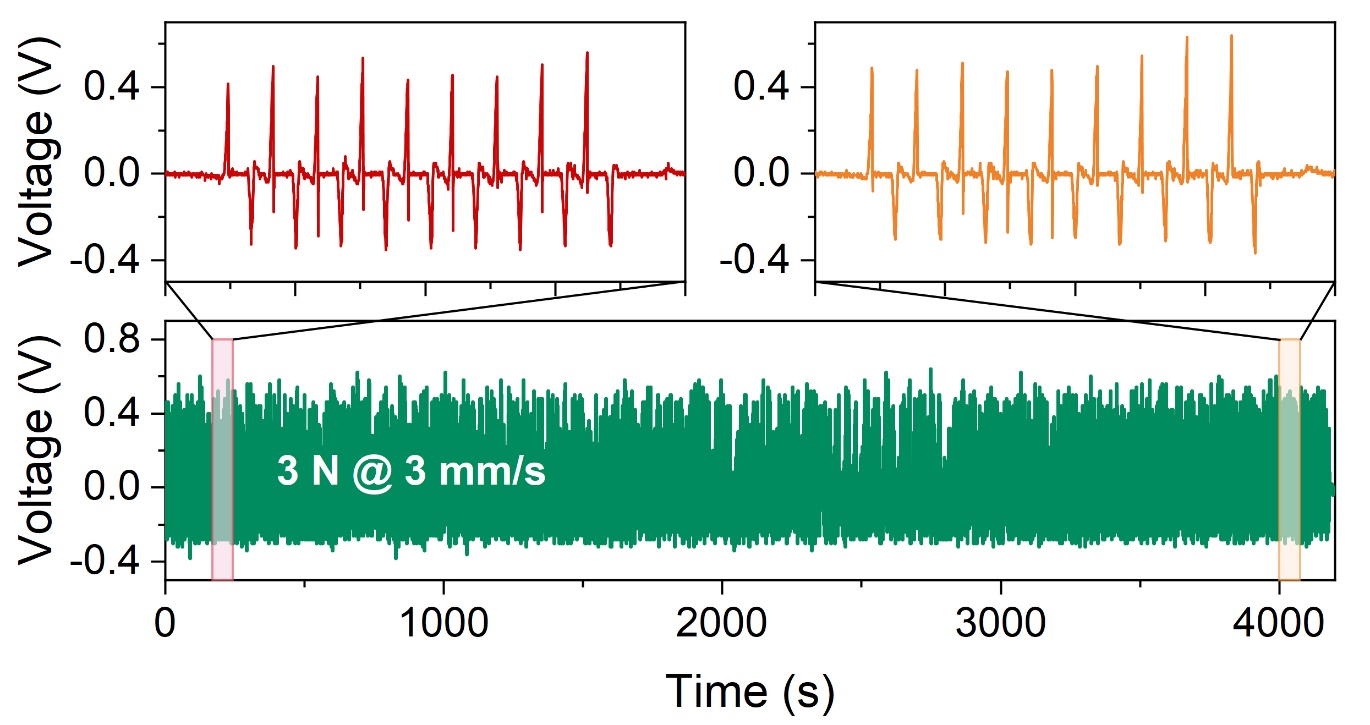


**Figure S15. Long-term durability test of SensorH6-Z over 5000 loading cycles under force of 3 N at the loading speed of 3 mm/s.**


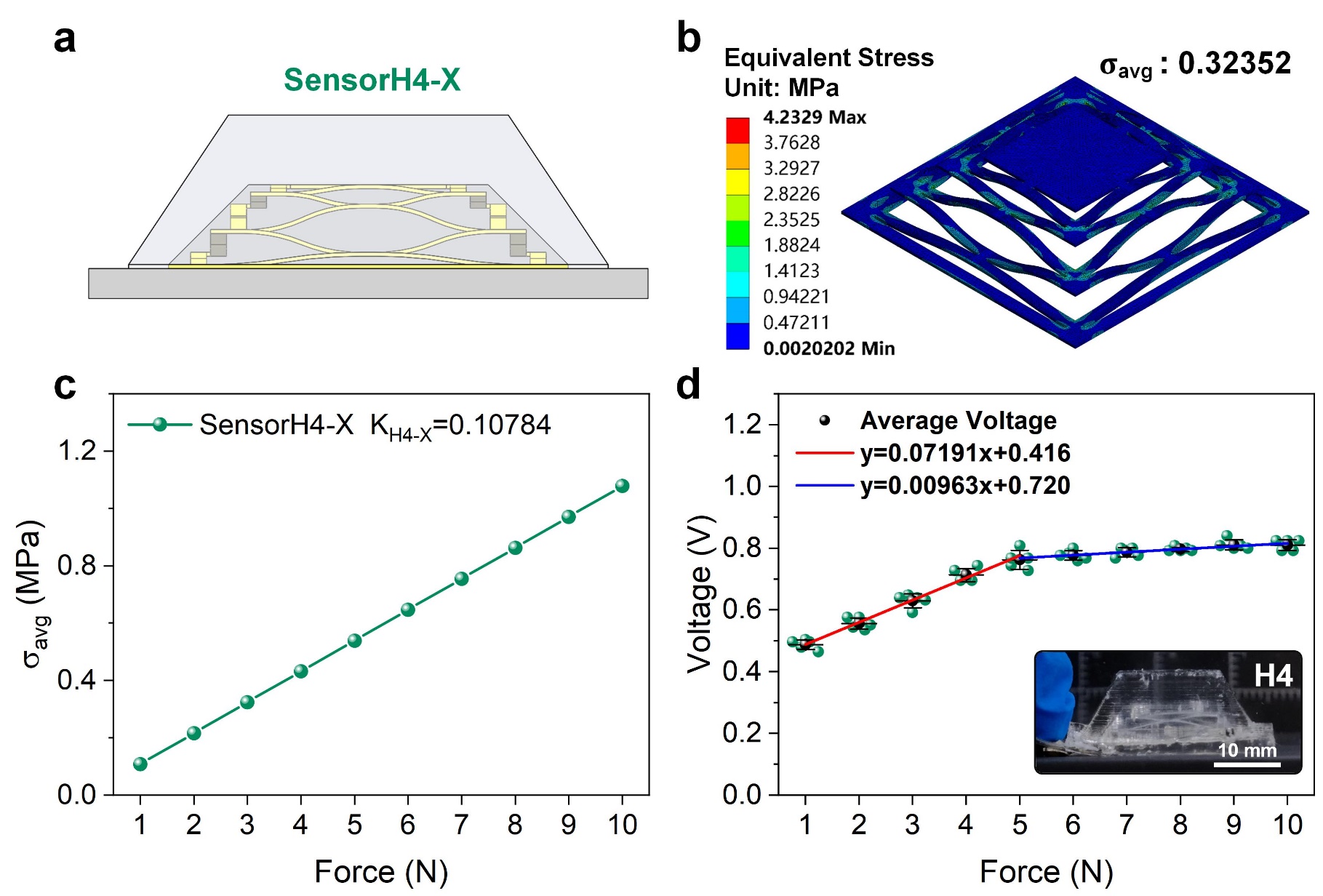


**Figure S16.** **(a)** Schematic illustrations of sensors implemented with deformed kirigami-cut PVDF films with displacement of 4 mm with pattern X (SensorH4-X). **(b)** Stress distributions of SensorH4-X according to FEA. **(c)** Numerical simulation results of SensorH4-X. **(c)** Measured output voltage as a function of applied normal force of SensorH4-X. The loading speed is 3 mm/s.


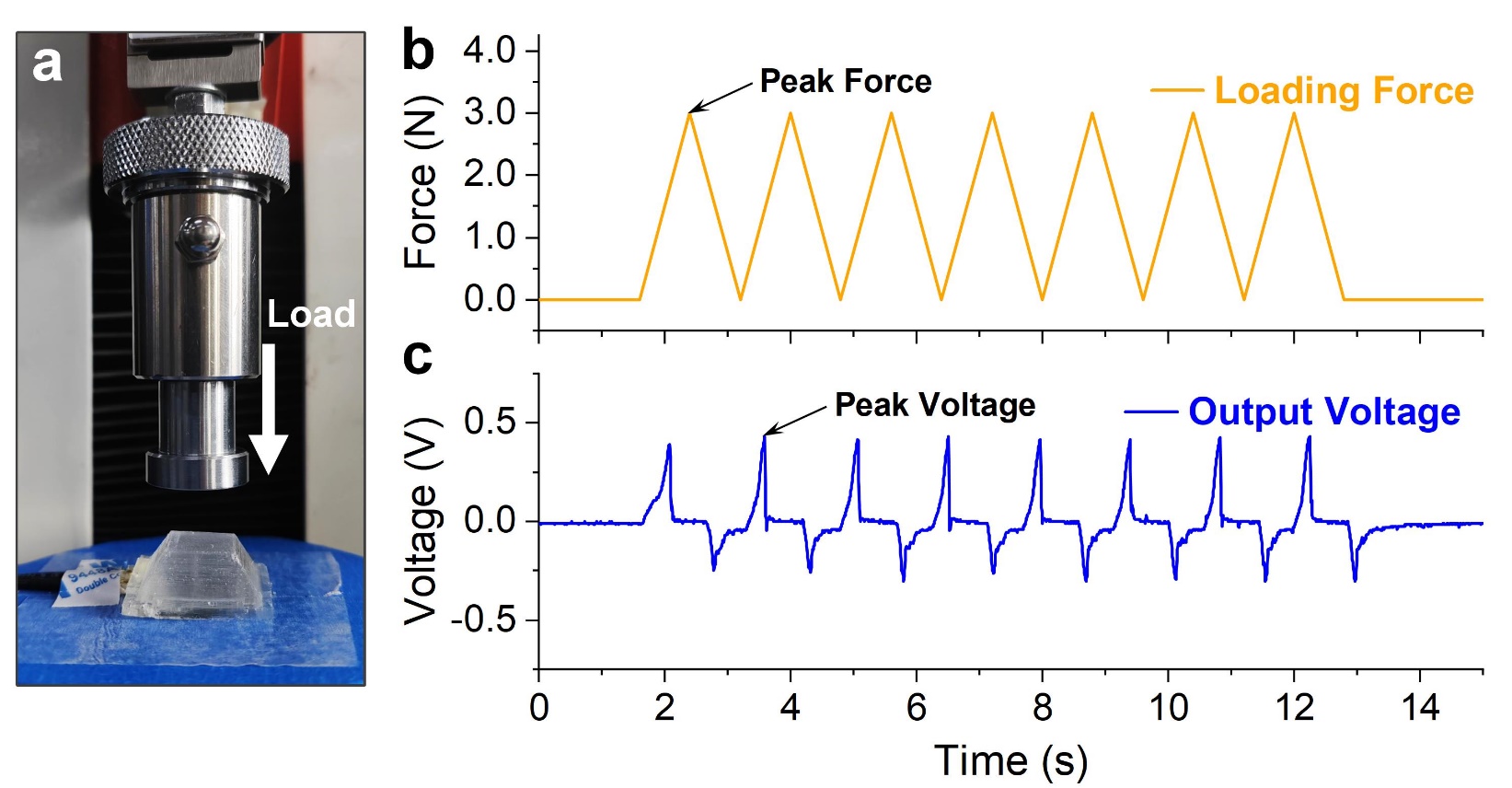


**Figure S17 . The experimental settings and cyclic loading procedure and typical output voltage signal.**

**Table S4.** Comparison of sensing performance between our sensor and existing PVDF-based piezoelectric sensors with structural design.

| **Material** | **Structure Design** | **Sensitivity** | **Force Range** | **Bandwidth** | **Ref.** |
| --- | --- | --- | --- | --- | --- |
| PVDF/ZnO nanofibers | Cowpea-structured | 0.33 V kPa^−1^ | 0.35–8.75 N | / | [1] |
| PDMS/PVDF/Silicone | rigid-soft hybrid | 346.5 pC N^−1^ | 0.009–4.3 N | 5–600 Hz | [2] |
| PVDF | dome-shaped | 6.028 × 10^−3^ V mN^−1^ | 0.1–1 N | 5 Hz | [3] |
| PVDF | kirigami cutting | 9.86 V/cm^2^ | 320.8% stretchability | / | [4] |
| PVDF | conductive sponge | / | 0.1–15 N | / | [5] |
| PDMS/PVDF/PDMS | Rigid-in-soft structure | 20.6 mV/N | 1–11 N | / | [6] |
| PDMS/PVDF | Deformed kirigami-cut PVDF | 0.08 V/N | 1-10 N | / | Our work |

**Reference:**

[1] W. Deng, T. Yang, L. Jin, C. Yan, H. Huang, X. Chu, Z. Wang, D. Xiong, G. Tian, Y. Gao, H. Zhang, W. Yang, *Nano Energy* **2019**, *55*, 516.

[2] J. Zhang, H. Yao, J. Mo, S. Chen, Y. Xie, S. Ma, R. Chen, T. Luo, W. Ling, L. Qin, Z. Wang, W. Zhou, *Nat. Commun.* **2022**, *13*, 5076.

[3] M.-S. Kim, H.-R. Ahn, S. Lee, C. Kim, Y.-J. Kim, *Sensors and Actuators A: Physical* **2014**, *212*, 151.

[4] Y.-G. Kim, J.-H. Song, S. Hong, S.-H. Ahn, *npj Flex Electron* **2022**, *6*, 52.

[5] Z. Wang, H. Leng, N. Li, T. Yao, *Adv. Mater. Technol.* **2022**, *7*, 2200260.

[6] X. Huang, Z. Ma, W. Xia, L. Hao, Y. Wu, S. Lu, Y. Luo, L. Qin, G. Dong, *Nano Energy* **2024**, *129*, 110019.
